# hTERT Increases TRF2 to Induce Telomere Compaction and Extend Cell Replicative Lifespan

**DOI:** 10.1111/acel.70105

**Published:** 2025-05-15

**Authors:** Nancy Adam, Yang Yang, Mahbod Djamshidi, Sara Seifan, Nicholas S. Y. Ting, Joel Glover, Nicolas Touret, Paul M. K. Gordon, K. V. Vineetha Warriyar, Hokan Krowicki, Christine Kim Garcia, Sharon A. Savage, Aaron A. Goodarzi, Duncan M. Baird, Tara L. Beattie, Karl Riabowol

**Affiliations:** ^1^ Robson DNA Science Centre, Arnie Charbonneau Cancer Institute, Departments of Biochemistry & Molecular Biology and/or Oncology, Cumming School of Medicine University of Calgary Calgary Alberta Canada; ^2^ Division of Cancer and Genetics, School of Medicine Cardiff University Cardiff UK; ^3^ Live Cell Imaging Laboratory, Cumming School of Medicine University of Calgary Calgary Alberta Canada; ^4^ Department of Biochemistry University of Alberta Edmonton Alberta Canada; ^5^ Centre for Health Genomics and Informatics, Cumming School of Medicine University of Calgary Calgary Alberta Canada; ^6^ Alberta Children's Hospital Research Institute, Cumming School of Medicine University of Calgary Calgary Alberta Canada; ^7^ Irving Medical Center Columbia University New York New York USA; ^8^ Clinical Genetics Branch, Division of Cancer Epidemiology and Genetics National Cancer Institute Bethesda Maryland USA

**Keywords:** hTERT noncanonical activity, senescence, telomere clustering, telomere compaction, TRF2 stabilization

## Abstract

Replicative senescence occurs in response to shortened telomeres and is triggered by ATM and TP53‐mediated DNA damage signaling that blocks replication. hTERT lengthens telomeres, which is thought to block damage signaling and the onset of senescence. We find that normal diploid fibroblasts expressing hTERT mutants unable to maintain telomere length do not initiate DNA damage signaling and continue to replicate, despite having telomeres shorter than senescent cells. The TRF1 and TRF2 DNA binding proteins of the shelterin complex stabilize telomeres, and we find that expression of different mutant hTERT proteins decreases levels of the Siah1 E3 ubiquitin ligase that targets TRF2 to the proteasome, by increasing levels of the CDC20 and FBXO5 E3 ligases that target Siah1. This restores the TRF2:TRF1 ratio to block the activation of ATM and subsequent activation of TP53 that is usually associated with DNA damage‐induced senescence signaling. All hTERT variants reduce DNA damage signaling, and this occurs concomitantly with telomeres assuming a more compact, denser conformation than senescent cells as measured by super‐resolution microscopy. This indicates that hTERT variants induce TRF2‐mediated telomere compaction that is independent of telomere length, and it plays a dominant role in regulating the DNA damage signaling that induces senescence and blocks replication of human fibroblasts. These observations support the idea that very short telomeres often seen in cancer cells may fail to induce senescence due to selective stabilization of components of the shelterin complex, increasing telomere density, rather than maintaining telomere length via the reverse transcriptase activity of hTERT.

## Graphical abstract

In contrast to the dogma that a subset of short telomeres initiates senescence, hTERT variants unable to maintain telomere length block senescence by stabilizing TRF2 via inhibiting upstream E3 ubiquitin ligases. This noncanonical hTERT function also compacts telomeres and blocks senescence‐associated DNA damage signaling. Cancer cells expressing hTERT may bypass senescence by maintaining TRF2 levels and telomere density to block DNA damage signaling and become immortal, even with very short telomeres.

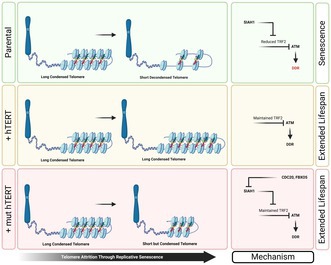

## 1 Introduction

Telomeres consist of (TTAGGG)_n_ DNA repeats and a telomere‐specific protein complex called shelterin that blocks single‐stranded G‐rich telomere ends from being recognized as damaged DNA (de Lange 2009). Shelterin proteins facilitate telomere (t)‐loop formation and block the DNA damage response (DDR) machinery via inhibition of individual proteins involved in the sensing and repairing of DNA breaks (Palm and de Lange 2008). In primary somatic cells, telomeres shorten with each cell cycle due to the end‐replication problem and end‐processing activities (Arnoult and Karlseder 2015; Harley et al. 1990). It is thought that telomere shortening, which coincides with the loss of the shelterin protein TRF2, leads to less efficient formation of the t‐loop structure, allowing telomeric DNA to be deprotected and recognized as a one‐sided double‐stranded break (DSB) (di d'Adda Fagagna et al. 2003). DSB recognition leads to activation of an ATM and TP53 signal transduction pathway to induce a permanent cell cycle arrest and replicative senescence (Atadja et al. 1995; Vaziri et al. 1997). Accumulation of senescent cells contributes to chronic inflammation, which creates a tissue environment that promotes the genesis of many age‐related diseases, including cancer.

In cancer cells, the expression of telomerase, a ribonucleoprotein (RNP) complex that is able to extend telomere length during the S‐phase of the cell cycle (Greider and Blackburn 1989), prevents replicative senescence, presumably by lengthening telomeres. In agreement with this idea, mutations in the human telomerase RNP, such as some in the telomerase reverse transcriptase protein (hTERT), lead to telomere shortening, premature senescence, and the development of several premature aging diseases, including dyskeratosis congenita (DKC) and idiopathic pulmonary fibrosis (IPF). IPF is a lung disease characterized by the loss of cell replication leading to scarring of lung tissue, while the DC phenotype includes PF as well as a range of complications, including bone marrow failure, liver disease, mucocutaneous abnormalities, and cancer predisposition (Garcia et al. 2007). Expression of two hTERT mutations associated with the onset of IPF (Tsakiri et al. 2007) was previously shown to be unable to maintain telomere length in primary fibroblasts. However, our group has found that despite a continuous loss of telomere sequence in cells expressing these mutants, cells continue to divide (Tsang et al. 2012), suggesting that additional factors regulate the initiation of senescence signaling.

Previous studies using single‐molecule localization microscopy (SMLM) have suggested that telomere compaction might affect senescence signaling through the effects of shelterin proteins on telomere compaction and their role in activating a DDR. While some studies reported that DDR factors were unable to access telomeric regions due to shelterin‐induced compaction (Bandaria et al. 2016), other studies found that shelterin removal did not significantly affect telomere compaction (Timashev et al. 2017; Vancevska et al. 2017) despite the induction of a DDR at most telomeres. These contrasting outcomes may be due to different SMLM techniques used, the exogenous manipulation of shelterin proteins, and/or the use of cancer cells expressing telomerase and mouse embryo fibroblasts (MEFs) that contain unusually long telomeres compared to normal human somatic cells. Therefore, it remains unclear whether telomere relaxation is required for the induction of a DDR and cellular senescence in diploid human cells.

Here, we present evidence that hTERT‐induced transcriptional regulation that increases TRF2 levels and telomere compaction, rather than telomere length, is the most proximal factor that inhibits telomere‐initiated DDR signaling and replicative senescence in human fibroblasts. This occurs independently of the senescence‐associated secretory phenotype (SASP). We show that diploid cells with telomere lengths similar to, or shorter than, those observed in senescent cells are able to undergo an extended replicative lifespan as a result of hTERT selectively regulating the expression of genes encoding proteins that localize to telomeres, resulting in telomere compaction and blocking of ATM‐mediated DNA damage signaling.

## 2 Results

### 2.1 Primary Diploid Fibroblasts Lose Similar Amounts of Telomeric DNA in Culture, Which Culminates in Cell Senescence

We first characterized one lung (WI38) and two dermal (Hs68 and BJ) primary fibroblast strains by passaging them in culture until they reached a state of replicative senescence. Growth curves (Figure S1A) and senescence‐associated beta‐galactosidase (SA‐β Gal) staining (Figure S1B) followed by determination of telomere length at different passage levels by telo‐qPCR (Figure S1C), indicated that all strains lost between 40 and 55 bp of telomeric DNA/telomere/cell division, in good agreement with rates reported previously using terminal restriction fragment (TRF) assays of normal human fibroblasts (Harley et al. 1990) and in agreement with standard curves done for telo‐qPCR assays (Figure S2).

### 2.2 Primary Fibroblasts Expressing hTERT Mutants That Do Not Elongate Telomeres Have Extended Replicative Lifespans

**FIGURE 1:**
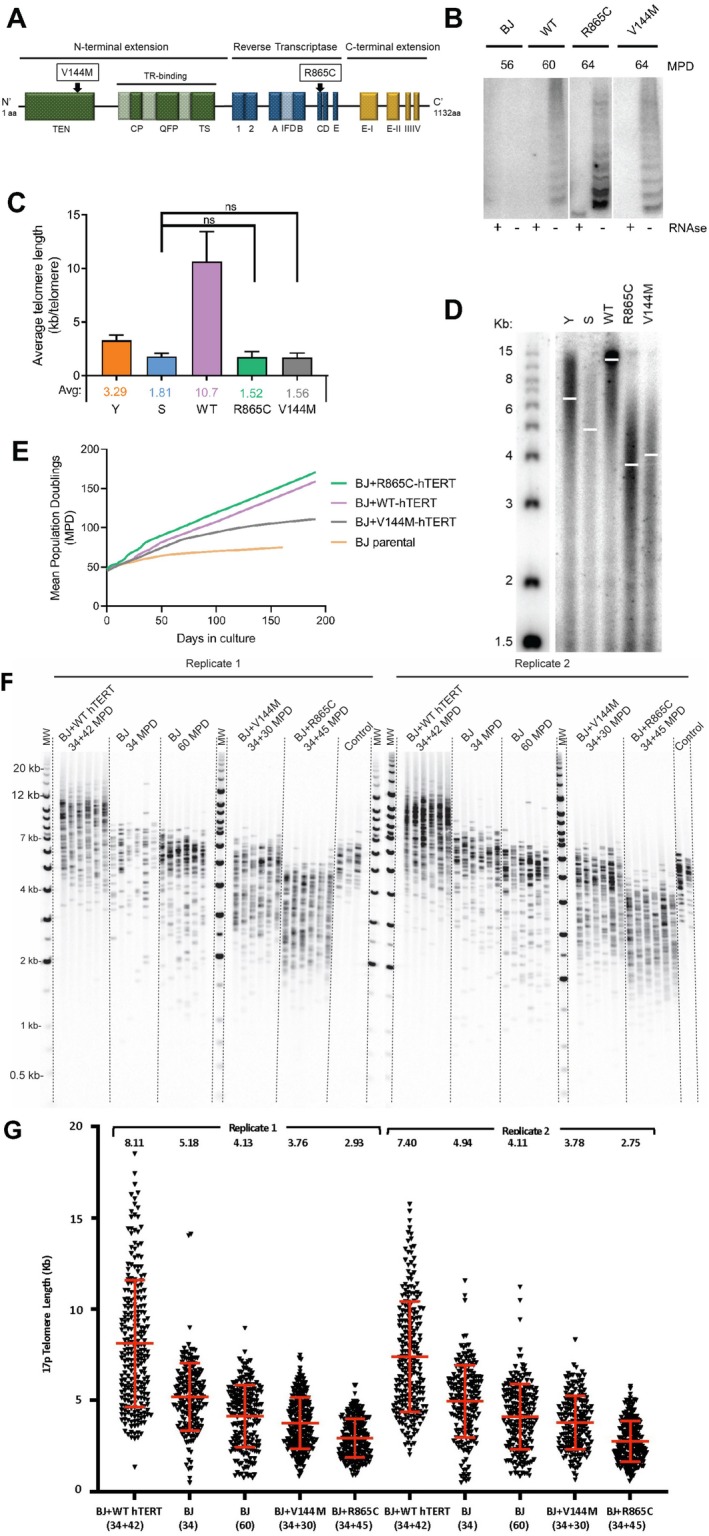
Catalytically active hTERT mutants do not extend telomeres in primary fibroblasts but do extend replicative lifespan. (A) Locations of V144M and R865C mutations identified in idiopathic pulmonary fibrosis patients in hTERT protein domains. (B) TRAP assay of the catalytic activity of WT‐, V144M‐, and R865C‐hTERT in vitro. (C) Average telomere length of young, senescent, and primary BJ fibroblasts expressing the indicated forms of hTERT after passage for 90 MPD as determined by telo‐qPCR. (D) TRF assay of telomere length in BJ young (~6.4 kb), senescent (~4.9 kb), +WT‐hTERT (~13.8 kb), +R865C‐hTERT (~4.0 kb), and +V144M‐hTERT (~4.1 kb) cells. White bars indicate average telomere length based on scanning the lanes and analysis of gels by scanning densitometry. (E) Growth curves of BJ fibroblasts (parental) and BJ cells expressing WT, V144M, and R865C mutants of hTERT. (F) Autoradiographs of the PCR products of STELA analysis for chromosome 17p of young and old BJ fibroblasts compared to STELA products of BJ fibroblasts overexpressing WT, V144M, and R865C versions of hTERT. Replicates 1 and 2 represent DNA samples isolated from separate plates of cells grown independently. The number of mean population doublings (MPDs) for cell samples is indicated. (G) Scatter plots of the telomere lengths of chromosome 17p in the mass cultures of BJ fibroblasts +/– hTERT variants showing means +/– standard deviations. Numbers above the plots indicate mean lengths. Numbers below the abscissa indicate the number of cell divisions each strain had undergone. Growth curves have been independently generated three times and showed consistent relative growth rates (*n* = 3).

Primary BJ dermal fibroblasts were stably transfected with wild‐type (WT) and mutant forms of the hTERT subunit of telomerase (Figure 1A) that in patients result in rare autosomal dominant hereditary disease characterized by early death due to pulmonary fibrosis and to aplastic anemia (Garcia et al. 2007; Tsakiri et al. 2007; Tsang et al. 2012). Cells expressing both WT and mutant hTERT showed catalytic activity in vitro (Figure 1B) but were unable to maintain telomere length. While cells expressing WT‐hTERT showed ~3‐fold longer telomeres after passaging in culture, those expressing mutant forms of hTERT showed telomere lengths as short as, or shorter than, senescent cells by both telo‐qPCR (Figure 1C) or TRF assays (Figure 1D). However, as reported previously (Tsang et al. 2012), cells expressing WT or mutant forms of hTERT proliferated faster and far beyond the replicative lifespan of control untransfected parental cells (Figure 1E). Since the ability to continue to proliferate despite an overall shorter average telomere length could be the result of selective elongation of a subset of the shortest telomeres in cells (Hemann et al. 2001), we next asked if expression of the mutant forms of hTERT were capable of elongating one of the shortest telomeres, even though the mean telomere length was significantly shorter than seen in senescent cells. The short arm of human chromosome 17 (17p) was found to have unusually short telomeres (Martens et al. 1998), and so we examined the length of chromosome 17p using single telomere length analysis (STELA) (Baird et al. 2003). As shown in Figure 1F, cells expressing mutants of hTERT had shorter ranges of 17p telomeres than senescent cells, most likely because they had undergone more cell divisions. As seen in Figure 1G, senescent WT BJ fibroblasts examined at 60 mean population doublings (MPDs) had longer 17p telomeres (4.13 and 4.11 in two biological replicates) than BJ fibroblasts expressing the R865C hTERT mutant (2.93 and 2.35 in two biological replicates) that had been growing for 34 + 45 = 79 MPDs. The two biological replicates of young and senescent cells and cells expressing the different forms of hTERT showed strong concordance, and the average relative lengths of telomere 17p in the different populations showed good agreement with telo‐qPCR and TRF assays of all telomeres. Cells expressing the WT form of hTERT that is effective at elongating telomeres showed markedly longer 17p telomeres compared to cells expressing mutant forms of hTERT that had undergone similar numbers of doublings. This confirmed that telomeres were not being elongated or selectively elongated by cells expressing high levels of mutant hTERT, as indicated by telo‐qPCR, TRF, and STELA assays.

### 2.3 Cells Expressing hTERT Mutants Have Fewer DNA Damage Foci Despite Having Short Telomeres

**FIGURE 2:**
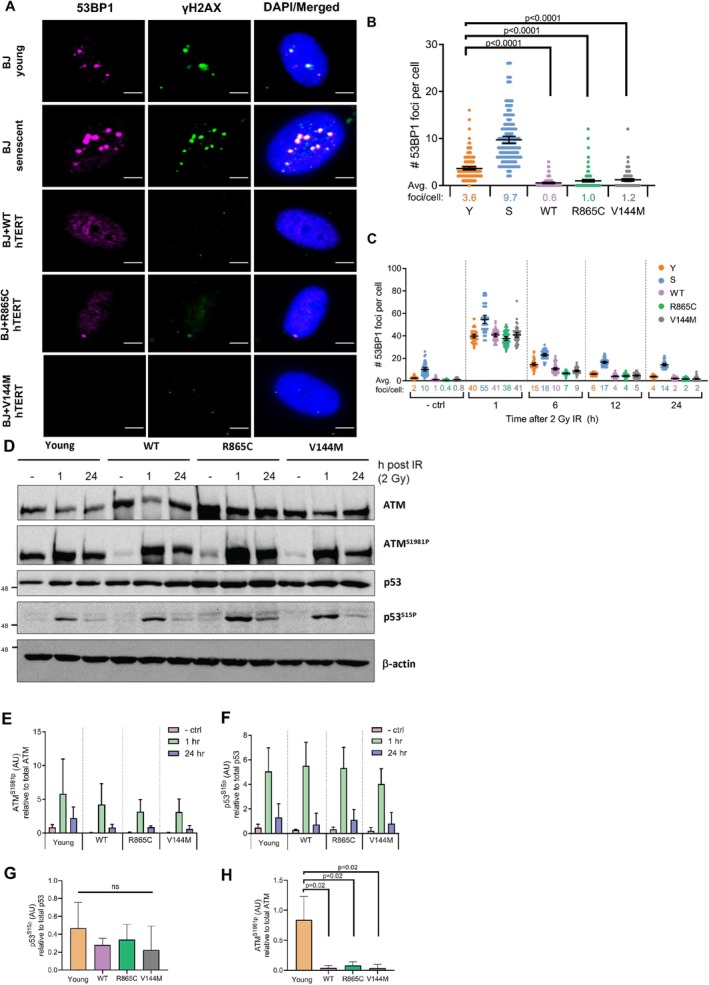
Cells expressing hTERT variants show reduced DNA damage signaling despite having short telomeres. (A) Representative wide‐field immunofluorescence images of 53BP1 (pink) and γH2AX (green) staining in BJ young, senescent, and BJ cells stably transfected with WT‐hTERT, R865C‐hTERT, and V144M‐hTERT (scale bar = 5 μm). (B) Quantification of the total number of 53BP1 foci per cell in resting cells based on wide‐field immunofluorescence images of 53BP1. Error bars = 95% CI; *n =* 3 with > 50 cells counted per *n; p* value determined by two‐way ANOVA. (C) Recovery rate based on counting numbers of 53BP1 foci in BJ young, senescent, and BJ cells stably transfected with WT‐hTERT, R865C‐hTERT, and V144M‐hTERT at the indicated time points after exposure to 2 Gy (IR). The control represents a replicate of the experiment shown in Panel b. (D) Western blot analysis of ATM, ATM^S1981p^, p53, and p53^S15p^ in young BJ cells and BJ cells stably expressing WT‐hTERT, R865C‐hTERT, and V144M‐hTERT before, 1, and 24 h after exposure to 2 Gy of g‐IR. (E) Quantification of ATM^S1981p^ protein levels after IR exposure. (F) Quantification of p53^S15p^ protein levels after IR exposure. (G) Quantification of basal p53^S15p^ protein levels relative to total p53 protein based on WB analyses as shown in panel a. (H) Quantification of basal ATM^S1981p^ protein levels relative to total ATM protein based on WB analysis in Panel A. (E–H) Error bars = 95% CI; *n =* 3; *p* values determined by two‐way ANOVA.

To test if cells with short telomeres but expressing hTERT mutants generated telomere dysfunction‐induced foci (TIFs), we stained young, senescent, and hTERT variant‐expressing BJ fibroblasts for the DDR factors 53BP1 and γH2AX in the absence of DNA damage as a baseline (Figure 2A). Senescent BJ fibroblasts had increased numbers of 53BP1 foci (9–10/cell) compared to young cells (3–4/cell), and cells expressing WT hTERT had markedly reduced numbers of DNA damage foci (Figure 2B). Despite having telomeres of a length similar to, or shorter than, senescent cells, cells expressing mutant forms of hTERT contained fewer DNA damage foci than young cells (1–2 foci/cell for mutants vs. 3–4 foci/cell for young cells). This is consistent with previous observations indicating that WT hTERT can enhance DNA repair and reduce spontaneous chromosome damage (Sharma et al. 2003) and inhibit TP53 activation and DNA damage signaling (Beliveau and Yaswen 2007). To test if hTERT expression blocked the formation of damage foci in response to DNA damage, cells were exposed to 2 Gray of ionizing radiation (2 Gy IR) and stained for 53BP1 foci before and 1, 6, 12, and 24 h after exposure. As shown in Figure 2C, all cells had a robust response to DNA damage, generating severalfold higher numbers of foci. Senescent cells showed reduced ability to repair foci by 24 h, and expression of hTERT variants reduced the number of DNA damage foci slightly more rapidly than seen in untransfected cells, since at 6 h postexposure, young cells had an average of 15 foci/cell while cells expressing the different hTERT variants had an average of 8–9 foci. Since the R865C and V144M mutants of hTERT were identified in patients with IPF that harbor one mutant and one WT allele of hTERT (Tsakiri et al. 2007) and DKC, where patients may have two mutated hTERT alleles (Niewisch et al. 2022), we next asked whether cells derived from IPF and DKC patients had altered DNA damage signaling. As shown in Figure S3, IPF and DKC cells showed higher levels of the DNA damage markers 53BP1 and γH2AX than cells from age‐matched unaffected siblings, indicating that when not overexpressed, mutant forms of hTERT did not inhibit the basal DDR.

### 2.4 hTERT Variants Inhibit Basal, but Not IR‐Inducible DNA Damage Signaling

Since cells expressing hTERT variants efficiently removed IR‐induced DNA damage markers, we asked whether ATM and its downstream target TP53 were activated normally in the presence of hTERT. As shown in Figure 2D–F, IR robustly induced the phosphorylation of both ATM and TP53 in the presence or absence of hTERT, indicating that the induction of a DDR was conserved in cells expressing hTERT variants. Under basal conditions in unirradiated cells, TP53 phosphorylation was modestly decreased (Figure 2G), which resulted in the preferential downregulation of TP53 target genes (Figure S3F,G, and Table S1). All forms of hTERT decreased the levels of phosphorylated ATM dramatically (Figure 2H), potentially explaining the reduction of DNA damage foci in unirradiated cells.

### 2.5 Expression of hTERT Mutants Stabilizes TRF2 Levels by Blocking Expression of the Siah1 TRF2 E3 Ubiquitin Ligase

**FIGURE 3:**
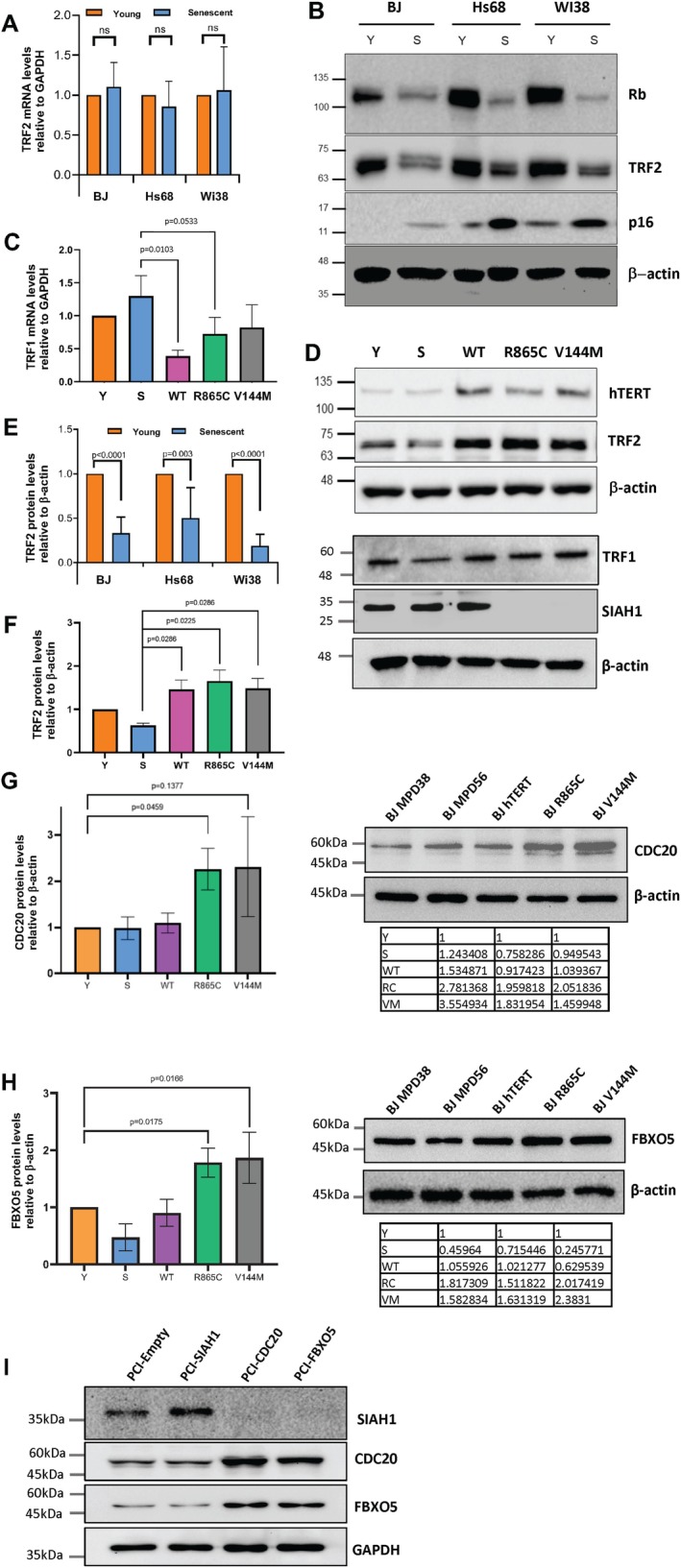
hTERT variants regulate the TRF2:TRF1 ratio by transcriptional and posttranslational mechanisms. (A) TRF2 mRNA levels determined by RT‐PCR in young and senescent BJ, Hs68, and Wi38 fibroblast strains. (B) Western blots of young (Y) and senescent (S) fibroblast strains probed with the indicated antibodies. (C) TRF1 mRNA levels in young and senescent BJ cells and BJs expressing WT‐hTERT, R865C‐hTERT, and V144M‐hTERT variants. (D) Western blots of young and senescent BJ fibroblasts, and BJ cells expressing hTERT variants, probed with the indicated antibodies. (E) TRF2 protein levels in young and senescent fibroblast strains. (F) TRF2 protein levels in young and senescent BJs and BJs expressing hTERT variants. (A,C,E,F) Error bars = 95% CI; *n* = 3. *p* values determined by two‐tailed unpaired *t*‐tests with Welch's correction, ns = not significant. (G) Quantitation of the levels of the CDC20 and (H) FBXO5 Siah1 e3 ubiquitin ligases in cells expressing hTERT variants. Graphs show three biological replicates with tables showing densitometry values for each replicate, with young BJ fibroblasts set to a value of 1. Error bars = 95% CI; *n* = 3. *p* values determined by two‐tailed unpaired *t*‐tests with Welch's correction. (I) Mock‐transfected cells or cells expressing Siah1, CDC20, or FBXO5 were harvested 48 h after transfection, and lysates were blotted for the indicated proteins. GAPDH served as the loading control.

Although cells expressing hTERT variants robustly responded to DNA damage, the activation of ATM was repressed in the absence of DNA damage, and it is known that TRF2 can affect the activation of DNA damage sensing by inhibiting ATM activation and suppressing the propagation of DNA damage signaling at the telomere (Karlseder et al. 2004). To ask if hTERT might inhibit ATM activation by altering TRF2 levels, we first measured TRF2 levels in young and senescent cells. As shown in Figure 3A, levels of TRF2 mRNA were not altered in senescing BJ, Hs68, or WI38 fibroblasts. Examination of protein lysates from the same cell preparations confirmed that senescing cells showed decreased levels of retinoblastoma (Rb) protein and increased levels of the p16 cyclin‐dependent kinase inhibitor, and also expressed reduced levels of TRF2 (Figure 3B), consistent with previous observations that senescent cells with activated TP53 (Atadja et al. 1995; Vaziri et al. 1997) induce Siah1‐mediated ubiquitination of TRF2 to reduce protein levels (Fujita et al. 2010; Mendez‐Bermudez et al. 2022). hTERT has not been reported to affect TRF2 levels. However, since TRF2 can delay senescence (Karlseder et al. 2002), we next asked if the expression of different forms of hTERT could alter levels of the two main shelterin proteins that directly bind DNA, TRF1 and TRF2. Unlike TRF2, hTERT variants slightly repressed TRF1 transcription (Figure 3C) but had little effect on TRF1 protein levels (Figure 3D). The reduced levels of TRF2 protein seen in all strains of senescent fibroblasts (Figure 3B,E) were elevated by all variants of hTERT, as shown in Figure 3D,F by ~2.5‐fold at the protein level. We next asked if hTERT variants that were unable to extend telomeres were stabilizing TRF2 levels by blocking Siah1‐induced TRF2 degradation. We noted that Siah1 protein was completely lost in response to mutant forms of hTERT expression (Figure 3D), explaining how cells with telomeres shorter than senescent cells maintained higher TRF2 levels. To ask if the reintroduction of Siah1 overcame the protective effects of hTERT mutants, we overexpressed Siah1 in BJ fibroblasts constitutively expressing the hTERT mutants and found that this induced DNA damage signaling at a level similar to that seen in senescent cells (Figure S4). Examination of Siah1 mRNA levels in our transcriptomic data indicated that the three hTERT variants had no consistent effect (WT –16%, R865C +3%, and V144M −12% vs. untransfected BJ fibroblasts), so we examined the mRNA levels of 20 candidate Siah1 E3 ubiquitin ligases listed in the Unibrowser database. Of these, the F‐Box protein 5 (FBXO5) and cell division cycle 20 (CDC20) genes were upregulated from 2.4‐ to 10‐fold by both the R865C and V144M hTERT mutants. Western blotting confirmed that both CDC20 and FBXO5 showed increased expression in cells expressing hTERT mutants (Figure 3G,H), consistent with their being responsible for inducing degradation of Siah1 to stabilize TRF2. To test this directly, we overexpressed Siah1, CDC20, or FBXO5, and 48 h later harvested cells for western blotting. As shown in Figure 3I, expression of both CDC20 and FBXO5 reduced Siah1 levels to well below those seen with the empty vector control.

### 2.6 hTERT Expression Blocks Telomere Clustering

Telomeres cluster in cancer cells that use the ALT mechanism for replication and that have long telomeres (Aten et al. 2004). Clustering is thought to occur in PML bodies that show characteristics of liquid–liquid phase separation (LLPS) (Draskovic et al. 2009). Using wide‐field microscopy, we previously found that long telomeres in WT‐hTERT‐immortalized fibroblasts also undergo a greater degree of clustering than in normal diploid fibroblasts with shorter telomeres (Adam et al. 2019).

**FIGURE 4:**
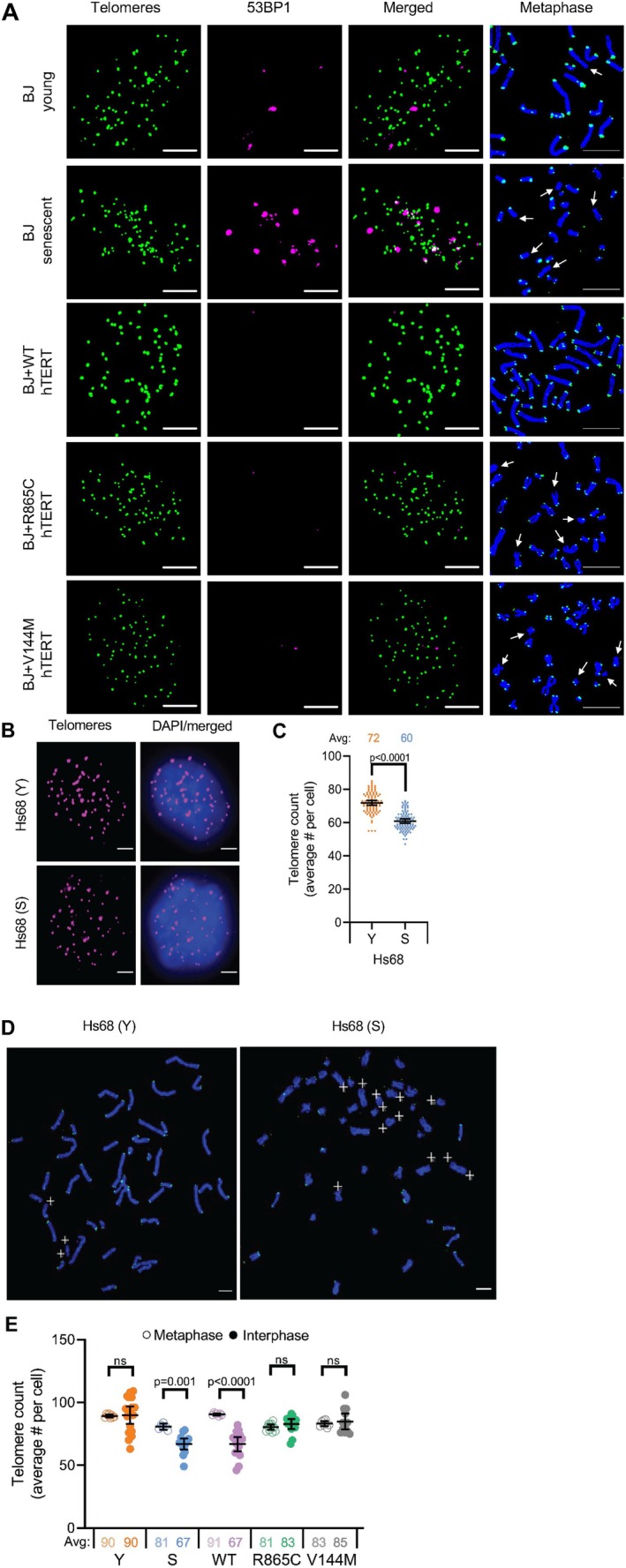
Super‐resolution imaging indicates that both long and dysfunctional telomeres cluster in interphase. (A) Representative structured illumination microscopy (SIM) immunofluorescence images of interphase BJ cells stained with PNA‐TelC488 probes (green), 53BP1 (magenta), merged telomeres with 53BP1, and corresponding metaphase spreads (DAPI = blue and telomeres = green). White arrows indicate telomere‐free ends (scale bar = 5 μm). (B) Representative wide‐field immunofluorescence images of telomere (magenta) staining in young (Y) and senescent (S) Hs68 cells in interphase (scale bar = 5 μm). (C) Quantification of telomere count per cell. Error bars = 95% CI; *n* = 3; *p* values determined by two‐tailed *t*‐tests with Welch's correction. (D) Metaphase spreads of Hs68 young (Y) and senescent (S) cells. Blue = DAPI and green = telomere staining. White crosses indicate telomere‐free ends (scale bar = 5 μm). (E) Quantification of the number of resolvable telomeres per cell based on interphase and metaphase cell staining in Panel D.

To ask if telomere clustering was affected by cell age or hTERT expression, we used super‐resolution structured illumination microscopy (SIM) and Airyscan microscopy, which have a lateral (*x,y*) resolution of 80–100 nm, to visualize telomeres. Analysis of young and senescent fibroblasts indicated that, as expected, senescent fibroblasts had more 53BP1 foci (Figure 4A) but fewer detectable telomeres in interphase (Figure 4B,C), due in part to an inability to visualize the shortest telomeres on some chromosomes, as also seen in metaphase spreads (Figure 4D). Examination of telomere counts in young and senescent BJ cells confirmed that fewer telomeres were detectable in senescent cells, but in contrast to young cells where identical numbers of telomeres were detected during interphase and mitosis (90 of the theoretically expected 92), many fewer telomeres were detected during interphase, suggesting that telomeres were colocalizing in senescent cells (Figure 4E). Cells that had very long telomeres due to expression of WT‐hTERT also showed reduced numbers of telomeres in interphase, consistent with our previous report of telomere clustering in primary fibroblasts expressing hTERT (Adam et al. 2019). In cells expressing the mutant forms of hTERT that had very short telomeres, no differences in the numbers of detectable telomeres were seen in mitotic versus interphase cells. Thus, although their telomeres were shorter than senescent cells, they showed no telomere clustering and few 53BP1 damage foci (Figure 4A), much as seen in young cells. These results suggest that deprotected telomeres cluster together in human senescent fibroblasts, resulting in a reduced telomere count/interphase cell. However, short telomeres in cells expressing hTERT mutants do not cluster, nor do they initiate a DDR, suggesting that 53BP1 expression may promote telomere clustering.

### 2.7 DNA Damage Foci Promote Clustering of Telomeres in Senescent Cells

**FIGURE 5:**
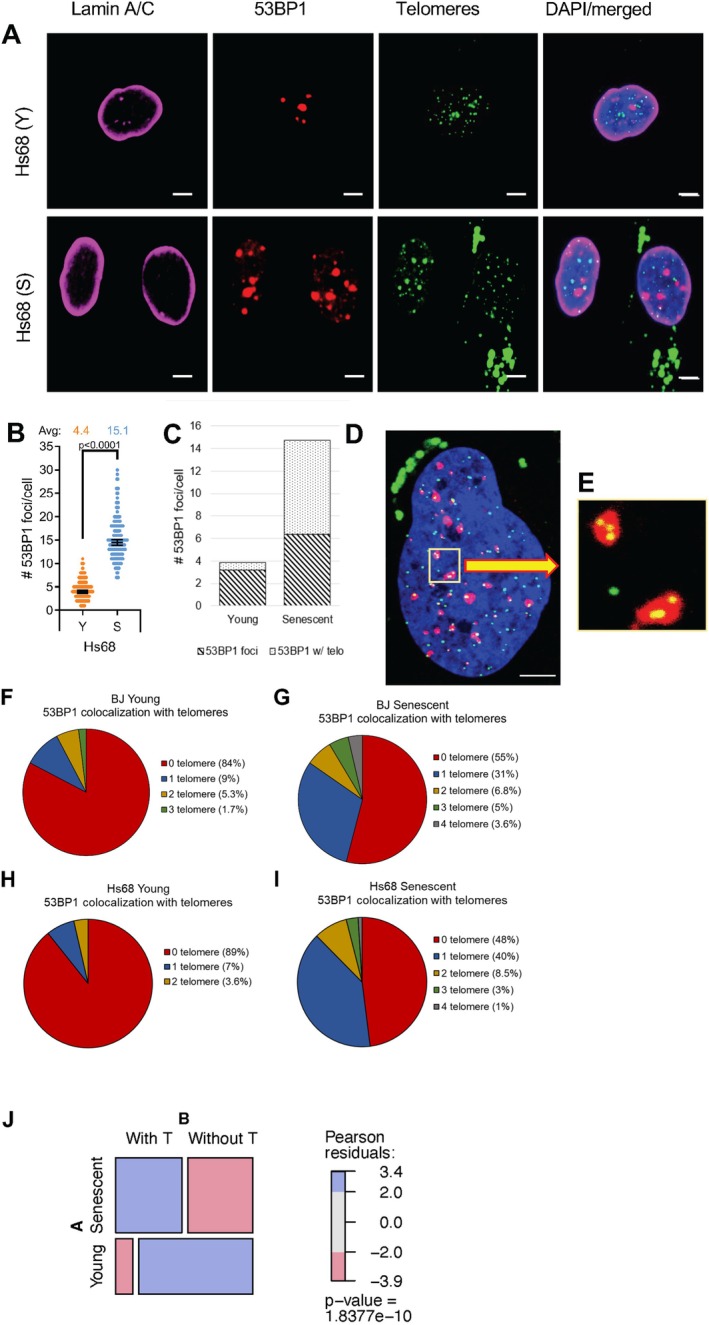
Multiple telomeres cluster in 53BP1‐foci in senescent cells. (A) Representative confocal immunofluorescence images of lamin A/C (magenta), 53BP1 (red), and telomere (green) staining in young (Y) and senescent (S) Hs68 cells (scale bars = 5 μm). (B) Quantification of the total amount of 53BP1 foci per cell. Error bars = 95% CI; *n* = 3 with > 50 cells counted per biological replicate. *p* values were determined by two‐tailed *t*‐tests with Welch's correction. (C) Quantification of the colocalization of 53BP1 with telomeres in young and senescent Hs68 cells. Over 50 cells were analyzed for young and senescent samples. (D) Airyscan image of a senescent Hs68 cell stained with PNA‐TelC488 probes (green) and 53BP1 (red) (scale bar = 5 μm). (E) Inset enlargement of the boxed area in (D shows clustering of two and three telomeres within each 53BP1 focus. Quantification of the numbers of telomeres that colocalize with 53BP1 foci in (F) young and (G) senescent BJ and (H) young and (I) senescent Hs68 fibroblasts. A total of 55–100 foci were examined for each cell type and age. (J) Pooled data for BJ and Hs68 fibroblasts showed that the increase in colocalization of 53BP1 with telomeres in senescent cells occurred in both strains and was significant with Pearson's *c* ^*2*^ = 40.63 and *p* = 1.84e‐10.

To better understand why cells with either long telomeres or short telomeres cluster their telomeres, we asked whether 53BP1 might be responsible for clustering. Damaged chromosome domains cluster (Aten et al. 2004) and 53BP1 localizes to sites of DNA damage (Ward et al. 2003) where 53BP1 oligomerization (Lottersberger et al. 2013) is thought to lead to LLPS to concentrate repair factors and promote more efficient DNA repair. To test whether the telomere clustering we observed in senescent cells was a result of increased levels of 53BP1, we investigated the number of telomeres that colocalized with a single 53BP1 focus using Imaris software as described (Bitplane, version 9.2.1) (Jeynes et al. 2017). As expected, senescent cells showed severalfold more 53BP1 foci (Figure 5A,B). Object‐based colocalization analysis showed that in young cells, 16% of 53BP1 foci colocalized with telomeres and 7% of foci colocalized with > 1 telomere (Figure 5C–E). In both senescent BJ and Hs68 primary fibroblast strains, these numbers increased ~3‐fold, with 45%–53% of 53BP1 foci colocalizing with telomeres and 13%–16% colocalizing with 2–4 telomeres (Figure 5F–I). This was highly significant (*p* = 1.84 × 10^‐10^, Figure 5J), indicating that clustering and colocalization of telomeres with 53BP1 damage foci normally occurs as cells senesce. To our knowledge, this is the first study using super‐resolution microscopy, which has sufficient resolution to quantify telomeres activating a damage response, to observe a significant increase in 53BP1‐associated telomere clustering in senescent cells.

### 2.8 Telomere Shape Does Not Affect DNA Damage Signaling

Since telomeres in cells expressing hTERT mutants are short, do not trigger a DDR, and do not cluster, and senescent cells exhibit enlarged, flattened nuclei that could affect telomere shape and compaction, we asked if telomere shape was different in cells expressing hTERT variants. Compacted telomeres have been proposed to be inaccessible for DDR factors (Bandaria et al. 2016), which could explain why these telomeres do not activate a DDR despite their short length. Using 3D direct stochastic optical reconstruction microscopy (dSTORM) and selection of telomere‐positive molecule clusters that were near the focal plane (Figure S7A), a variety of telomere shapes that had different radii of gyration (Rg) were observed (Figure S7B–E). Of note, irregular and bridged telomeres that may indicate telomere clustering were seen in both senescent and WT‐hTERT‐immortalized cells. The ratios of *x*, *y*, and *z* radii did not always correlate with the Rg or the observed shapes, indicating that large telomeres (those with large Rg values) were not always indicative of irregular‐shaped or bridged telomeres (Figure S7B–E). These results show that telomeres in both senescent cells and in cells expressing hTERT mutants are heterogeneous in shape and radius, and so shape is unlikely to be a factor in blocking the generation of a senescence signal from short telomeres.

### 2.9 Senescent Cells Contain Relaxed Telomeres

**FIGURE 6:**
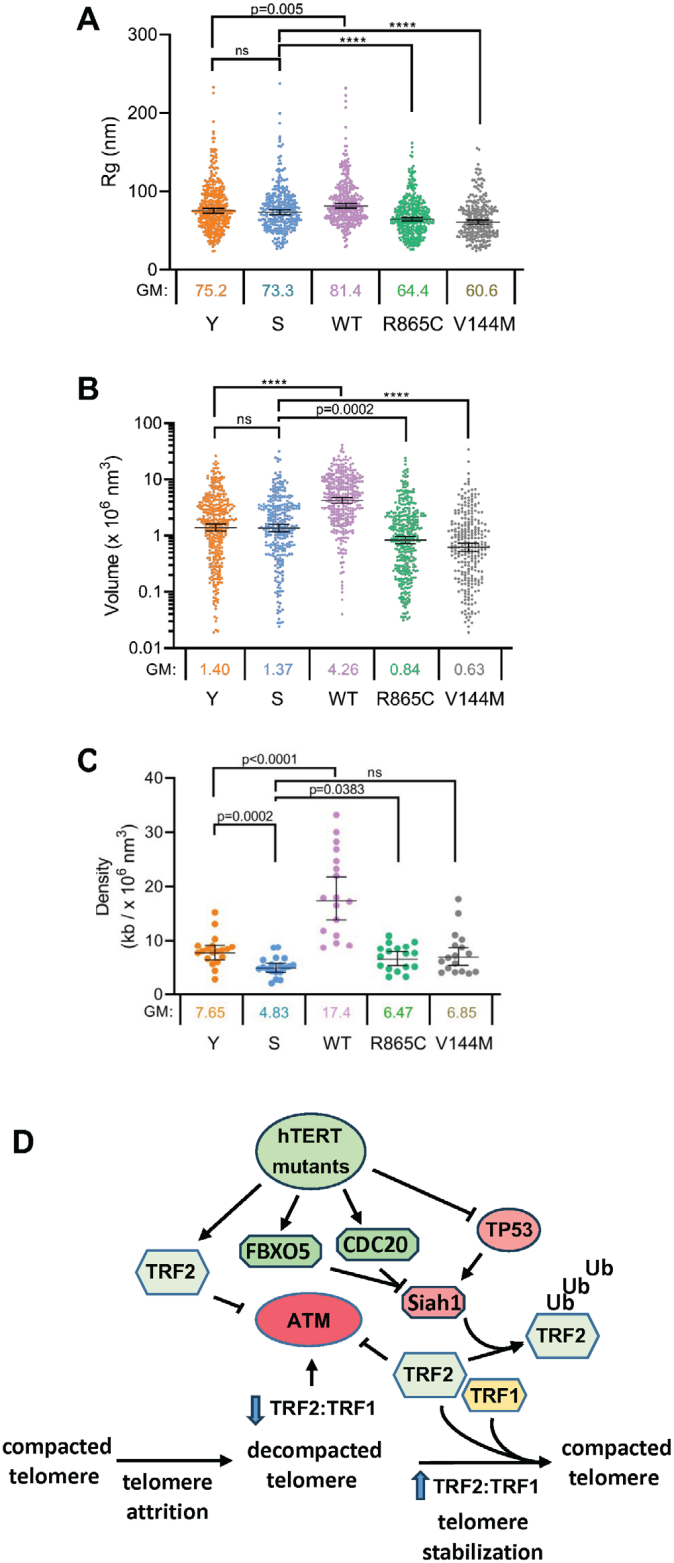
hTERT variants increase telomere density. (A) Radius of gyration (Rg) and (B) volume of telomeres in BJ young, senescent, and in BJ cells expressing WT‐hTERT, R865C‐hTERT, and V144M‐hTERT. GM = geometric mean, error bars = 95% CI; *n* = 3 with > 50 cells counted per biological replicate. (C) Density measurements based on average telomere length in kb and Rg^3^ with senescent cells showing the least dense telomeres (4.83 kb/10^6^ cubic nm). *p* values determined by one‐way ANOVA. ****< 0.001. (D) hTERT variants regulate the TRF2:TRF1 ratio to regulate telomere density and block telomere‐induced senescence signaling. TP53 activity increases (Atadja et al. 1995; Vaziri et al. 1997) and TRF2 levels drop significantly in all strains of normal diploid fibroblasts undergoing senescence that were examined. Expression of hTERT variants causes TRF2 levels to increase due to loss of the Siah1 TRF2 E3 ubiquitin ligase protein that is tagged for degradation by the FBXO5 and CDC20 Siah1 ubiquitin ligases that are transcriptionally induced by hTERT variants. TP53 activity that induces Siah1 expression (Fujita et al. 2010) decreases and also contributes to stabilizing TRF2. This increases TRF2 protein levels severalfold higher than seen in senescent cells to increase the TRF2:TRF1 ratio to levels seen in young, replication‐competent cells. Telomere‐bound TRF2 promotes telomere compaction, which inhibits activation of ATM to block damage signaling, p53 activation, and 53BP1 focus formation at telomeres.

To compare the compaction state of long and short telomeres, we measured the Rg and volume of individual telomeres in young, senescent, and hTERT‐expressing cells. Rg better represents overall telomere size independent of its shape, since this measurement averages the distance of each individual molecule to the centroid of the point cloud representing a telomere. Volume measurements are more sensitive to the outer points of the molecule cluster and can indicate changes in the size and shape based on the boundary of the telomere. In all three fibroblast strains, the average Rg values of the young cells were 72–75 nm, while Rg measurements in senescent cells were only slightly lower (Figure 6A). The same trend was observed in the volume measurements (Figure 6B). In order to calculate telomere density, we divided average telomere length (*TL*) measured by q‐PCR by the (Rg) (Harley et al. 1990) using the formula $\text{density}=\frac{\mathrm{TL}}{{\left(R_{g}\right)}^{3}}=\frac{\mathrm{kb}}{{\mathrm{nm}}^{3}}*10^{6}$. Plotting the average density of telomeres in individual cells indicated that senescent cells contained significantly less dense telomeres than young cells (Figure 6C), indicating that the DNA of short telomeres in senescent cells is of a more relaxed (i.e., less dense) nature.

### 2.10 Cells Expressing hTERT Variants Have Dense, Compact Telomeres

Cells expressing WT‐hTERT had larger telomeres as estimated by Rg than young cells, as expected, since they have ~3‐fold more telomeric DNA/cell (Figure 1C,D). This threefold increase in telomere length is reflected in volume measurements, where average WT‐hTERT cell telomere volume is ~4.26 × 10^6^ nm^3^ while young cell telomere volume is ~1.40 × 10^6^ nm^3^ (Figure 6B). Cells expressing hTERT mutants had significantly smaller telomeres as estimated by Rg when compared to senescent cells (Figure 6A), despite hTERT mutant strains and senescent cells having similar telomere lengths. This suggested that cells expressing mutant hTERT contain denser (i.e., more compact) telomeres.

To estimate the degree of telomere compaction using an independent approach, we used genomic digestion with micrococcal nuclease (MNase), a small enzyme (17 kDa) capable of cleaving nucleosome linker DNA that has relatively greater nuclease activity at more relaxed chromatin versus compacted chromatin (such as heterochromatin), followed by Southern blotting and hybridization with ^32^P‐labeled telomeric probe. MNase digestion generated a ladder of bulk nucleosomes in all samples examined and showed DNA fragments of sizes consistent with mono‐, di‐, tri‐, and larger polynucleosomes after 8 min incubation, indicating advanced genomic digestion (Figure S8A,B). WT‐hTERT telomere DNA was the most resistant to MNase digestion, showing reduced digestion products consistent with more compact chromatin with less accessible linker DNA after 4‐ and 8‐min incubation, while more complete digestion patterns were seen in other samples. Senescent cell telomeres were most sensitive to MNase digestion and showed almost complete digestion after 4 min (Figure S8A,B). Cells expressing mutant hTERT had telomeres that were more resistant to MNase digestion than senescent cells, which is evident by the lateral shift at the 4‐min time point (Figure S8). Thus, the order of telomere density seen was WT‐hTERT>young cells>V144M>R865C>senescent cells, consistent with telomere density regulating the activation of a DDR.

### 2.11 ATM Does Not Affect Telomere Compaction in Senescent Cells

Ataxia telangiectasia mutated (ATM) protein kinase promotes relaxation of heterochromatin to facilitate DNA double‐strand break repair and promotes transduction of the DDR in senescent cells (Vaziri et al. 1997). Since telomeric DNA is heterochromatic, we treated cells with the ATM inhibitor KU‐55933 to ask whether ATM might be responsible for decreasing telomere compaction in senescing cells. Staining cells for the ATM substrate KAP1^S824p^ 30 min after exposure to 2 Gy of γ‐ray IR (Goodarzi et al. 2008) confirmed inhibition (Figure S9), with a strong reduction in γH2AX foci signal intensity. However, little if any effect was seen on Rg in response to KU‐55933, indicating that ATM protein kinase activity does not function upstream of telomere‐initiated DDR nor induce telomere relaxation in senescent cells.

### 2.12 The SASP is Not Regulated by hTERT Variant Expression

The SASP is a marker of replicative senescence and can also be induced by oncogenic ras and inactivation of TP53 (Coppé et al. 2008). It is initiated by the recognition of cytoplasmic chromatin fragments by cGAS, resulting in the production of 2’3’cGAMP. This cyclic dinucleotide activates the STING‐NF‐κB and TBK1‐IRF3 pathways to induce expression of a characteristic set of genes, including cytokines like IL6 and IL8. Since cytoplasmic chromatin fragments could be generated by many mechanisms, such as DNA damage, by extrachromosomal DNA containing highly repetitive elements such as L1 that increase with age (Riabowol et al. 1985) or by degradation of a subset of mitochondria (Victorelli et al. 2023) that may be affected by hTERT, we asked how the expression of different hTERT proteins affected the transcriptome and whether they would affect the magnitude and/or composition of the SASP. BJ fibroblasts at low (young) and high (old) passage levels and BJ cells expressing WT, V144M, or R865C forms of hTERT were grown to log phase, and three sets of RNA were harvested for each strain independently. Following reverse transcription and cDNA library production, RNAseq was performed. As shown in Figure S5, Ingenuity Pathways Analysis (IPA) showed that the replicates were very similar and that principal component analysis of cells expressing hTERT variants showed that they had distinct transcription profiles that were clearly separable from both young and old strains of BJ fibroblasts. This is consistent with a previous study that reported distinct gene expression profiles of BJ cells and of BJ cells expressing hTERT (Lindvall et al. 2003). Cells expressing the WT form of hTERT that have undergone the greatest number of population doublings had transcriptomes similar to old, senescing fibroblasts despite having the longest telomeres of the different sets of cells examined. This suggests that maintenance of telomere length and replication ability does not block the effects of replicative lifespan on the expression of those genes comprising the major contributions to PCA analysis. Genes contributing most strongly to PCA scores included two forms of collagen (NM_000089 and NM_000088 encoding collagen type 1 α1 and α2 chains [COL1A1 and COL1A2]), eukaryotic translation elongation factor 1 α1 (NM_001402 [EEF1A1]), actin β (ΝΜ_001101 [ΑCTΒ]), and thrombospondin 1 (NM_003246 [THBS1]). Examination of the transcriptomes of WT BJ cells and BJ cells expressing different hTERT variants indicated that similar proportions of genes were upregulated (Figure S4B) or downregulated (Figure S4C) by hTERT, with ~10% of genes being commonly upregulated and 10% commonly downregulated by all hTERT variants. Senescent BJ fibroblasts expressed 51 of 79 previously described SASP gene products (Coppé et al. 2008) secreted by fibroblasts. When the transcriptomes of cells expressing WT or mutant forms of hTERT were examined, SASP genes were also upregulated (Figure S5D) or downregulated (Figure S5E), but, like total gene expression, the subsets varied between cell strains expressing different forms of hTERT, and similar numbers of SASP genes were up‐ and downregulated with little overlap in the subset of SASP genes regulated by different hTERT variants. This indicates that the expression of hTERT variants that can block the DDR did not affect SASP signaling by a common mechanism, suggesting that telomere‐initiated DNA damage signaling is not coordinately regulated with the SASP response in BJ human diploid fibroblasts.

### 2.13 hTERT Variants Inhibit TP53 Target Gene Expression

Expression of all hTERT variants blocked basal ATM^S1981^ phosphorylation, and to a lesser extent TP53^S15^ phosphorylation, which are indicators of ATM and TP53 activation (Figure 2). To ask if expression of the hTERT variants affected TP53 target gene expression, we examined our transcriptomic data for expression of genes previously identified as TP53 targets (Fischer 2017). Although 11% of TP53 target genes were induced by all hTERT variants (Figure S5F), more than twice as many (23%) were downregulated (Figure S5G), suggesting that all the hTERT variants had an overall inhibitory effect on TP53 signaling, as reported previously for WT hTERT (Beliveau and Yaswen 2007). The list of TP53‐responsive genes affected by hTERT variants is shown in Table S1.

### 2.14 hTERT Variants Regulate a Gene Set Affecting Telomere Biology

Transcriptomic data also indicated that genes that affect the levels and function of TRF2 were regulated by hTERT variants. Transcription of the Siah1 ubiquitin ligase that targets TRF2 for degradation (Fujita et al. 2010) was not altered, but mutant hTERT expression resulted in complete elimination of the Siah1 TRF2 E3 ubiquitin ligase protein (Figure 3D). This was due to transcriptional induction by the mutant hTERT variants of FBXO5 and CDC20 that serve as E3 ubiquitin ligases for Siah1, as shown directly (Figure 3G,H). Siah1 loss explains the increase of TRF2 protein in response to hTERT variants, which, together with the observation that TRF2 can condense chromatin more effectively than TRF1 (Poulet et al. 2012), likely underlies the hTERT‐driven increase in telomere density we find, since TRF2 interacts with histones, stabilizing chromosome ends in telomeres and inducing telomere compaction (Baker et al. 2011). Once localized at telomeres, TRF2 recruits ORC1 (increased transcription of 13‐87‐fold by all hTERT variants) that promotes heterochromatization and genomic stability (Higa et al. 2021). Increased TRF2 inhibits the activation of ATM (Karlseder et al. 2004) to block the formation of 53BP1 foci (Figure 2A,B) that normally promote nonhomologous end joining (NHEJ) (Dimitrova et al. 2008). These observations confirm reports that hTERT affects a subset of genes (Ye et al. 2014), and extend this idea to include hTERT mutants unable to elongate telomeres.

## 3 Discussion

In this study, we confirm that hTERT mutants that are unable to extend telomeres significantly extend proliferative lifespan and present evidence that longer telomeres in untransformed somatic mesenchymal fibroblasts promote telomere clustering. The transcriptional activity of TP53 decreases in cells expressing hTERT variants, as noted previously for WT‐hTERT (Beliveau and Yaswen 2007) and hTERT mutants, which destabilize the Siah1 E3 ubiquitin ligase that targets TRF2 to the proteosome via the transcriptional induction of the FBXO5 and CDC20 proteins that serve as Siah1 E3 ubiquitin ligases. Stabilized TRF2 inhibits ATM to prevent DNA damage signaling and compacts telomeres into higher‐density structures. We also find that senescent cell telomeres, which tend to cluster and colocalize with the DDR marker 53BP1, are in a less dense, relaxed state that facilitates the generation of the damage‐associated senescence response. These observations suggest that if short telomeres are protected and contain telomeric chromatin of a compacted nature, DDR‐initiated senescence is blocked. In the case of precancerous cells, expression of telomerase would allow further cell proliferation to promote genomic instability and tumorigenesis, even though telomeres were relatively short, as previously reported for many cancer types (Counter et al. 1992).

No telomere clustering was observed in BJ cells expressing hTERT mutants despite shortened telomeres. This suggests that clustering of short telomeres occurs when they display DNA damage that recruits 53BP1 to the telomeres to initiate clustering. This is consistent with 53BP1‐mediated telomere clustering stabilizing deprotected telomeres in senescent cells.

This study presents further evidence that chromosomes with long telomeres undergo clustering in an hTERT‐immortalized, but otherwise normal, cell background. While many studies have reported that telomeres associate with each other, the biological function of telomere clustering remains unknown, but may also be driven by LLPS. Since cells expressing either V144M‐ or R865C‐hTERT show more resolved telomeres in interphase than WT‐hTERT (Figure 4E), it is unlikely that hTERT protein alone mediates telomere clustering, but may be mediated by gene products commonly regulated by hTERT variants.

Based on dSTORM data, senescent cells contained less dense telomeres than young cells in BJ, WI38, and Hs68 strains, and MNase digestion was consistent with the accumulation of nucleosome‐free regions in short telomeres. Nucleosomes affect many DNA‐dependent processes by inhibiting the access of regulatory proteins, including those involved in transcription and repair. Both dSTORM and MNase experiments consistently showed that short telomeres in cells expressing hTERT mutants were more compact than in length‐matched senescent cells. This suggests that hTERT increases telomere density by affecting nucleosome arrangement, most likely via stabilizing TRF2, which alters nucleosome structure and induces chromatin compaction (Poulet et al. 2012), resulting in the protection of short telomeres and preventing the initiation of a DDR.

Shelterin proteins alter DNA structure and topology, condensing telomeric DNA in vitro. One study, using PALM and HeLa cells, investigated the role of shelterin proteins on telomere compaction in vivo and reported that TRF1 and TRF2 depletion resulted in the decompaction of telomeres by ~10‐fold (Bandaria et al. 2016). However, two subsequent studies using STORM and a variety of different cell lines reported that depletion of TRF2 only induced a modest increase in telomere volume and was not required for DDR signaling (Timashev et al. 2017; Vancevska et al. 2017). Our data from normal diploid human fibroblasts show that hTERT variants increase TRF2 levels, decrease TRF1 levels, and increase telomere density, concurrently with blocking the activation of ATM and the formation of DNA damage foci. Tethering of heterochromatin protein HP1α to telomeres was also reported to induce formation of large, irregular telomeres, reduce telomere damage, and increase telomere protection (Chow et al. 2018), consistent with our observations of hTERT variants increasing telomere density and blocking DNA damage signaling. Unlike WT hTERT, the hTERT mutants appear to strongly affect the levels of Siah1, CDC20, and FBXO5 to regulate TRF2 levels. It will therefore be interesting to determine whether the mutants that block telomere elongation despite being able to promote telomere density and block DNA damage signaling localize to telomeres in a way similar to WT hTERT.

Our data also highlight two independent mechanisms by which telomeres may be induced to cluster: by DNA damage‐induced clustering associated with 53BP1 protein foci, and by physical processes associated with telomere length, most likely involving telomere‐induced LLPS. These data also support the hypothesis that the hTERT protein, and other forms of hTERT that are ineffective in elongating telomeres in cells, inhibit TRF1 transcription and stabilize TRF2 protein to alter telomere compaction, thereby blocking the DDR and allowing replication. Thus, in contrast to the theory that telomere length itself serves to induce the process of replicative senescence, the more immediate event promoting the senescence phenotype appears to be TRF2:TRF1 ratio‐mediated telomere structure, which may explain how many cancer cells appear to flourish with significantly shorter telomeres than normal somatic cells (Xu and Blackburn 2007) while displaying significant genomic instability.

## 4 Experimental Procedures

### 4.1 Cell Lines

Primary Hs68 human foreskin fibroblasts (ATCC CRL‐1635) and WI38 fetal lung fibroblasts (ATCC CCL‐75) were grown in DMEM, 1 g/L glucose (Gibco, 11885‐084), supplemented with 10% (v/v) FBS. Human BJ foreskin fibroblasts (ATCC CRL‐2522) were grown in EMEM (Gibco) supplemented with 10% (v/v) FBS. Cells were grown at 37°C, 5% CO_2_ in humidified incubators. All cell cultures were tested bi‐weekly for mycoplasma and were uniformly negative. BJ cells expressing hTERT variants were isolated after infection with empty retrovirus (pBABEpuro) or with pBABEpuro containing the hTERT variants in the presence of polybrene, selected in puromycin, and characterized for replication potential as described in Tsang et al. (2012)). The NCI‐422‐1 (p.V664M) primary fibroblasts were derived from a 9‐year‐old male participant in an IRB‐approved inherited bone marrow failure syndromes study (ClinicalTrials.gov Identifier NCT00027274) after informed, written consent from the parent.

### 4.2 Telomere Length Analysis via qPCR

To analyze telomere length, genomic DNA (gDNA) was extracted from cell pellets using the QIAamp DNA Mini Kit (QIAGEN, Cat No. 51304) following the manufacturer's protocol. The concentration of gDNA was measured using a NanoDrop 2000 (Thermo Scientific). Each q‐PCR reaction contained 1× PowerUp SYBR Green Master Mix (Applied Biosystems, A25742), 20 ng gDNA, 0.1 μM forward primer, and 0.1 μM reverse primer. The following PCR cycles were used: hold stage: 95°C, 10 min; PCR stage (40 cycles): 95°C, 15 s–60°C, 1 min; and melt curve stage: 95°C, 15 s–60°C, 15 s–95°C, 15 s. For absolute telomere length measurements, a standard curve was generated by diluting known quantities of synthesized oligos of TTAGGG repeats and a 36B4 sequence (single‐copy gene control) (Figure S2). The line equation of the standard curve was used to determine the amount of TTAGGG and 36B4 of each sample based on the measured *Ct* values. The absolute telomere length per chromosome was calculated according to: $\text{Average telomere length}=\left(\frac{10^{\mathrm{LOG}\left[36B4\;\text{copies}\right]}}{10^{\mathrm{LOG}\left[\mathrm{TL}\;\left(\mathrm{kb}\right)\right]}}\right)/92$.

Primer sequences used for telomere length measurements:

**Table:** 

Label	Sequence 5′‐3’
Telo‐Forward	CGGTTTGTTTGGGTTTGGGTTTGGGTTTGGGTTTGGGTT
Telo‐Reverse	GGCTTGCCTTACCCTTACCCTTACCCTTACCCTTACCCT
Telo standard	(TTAGGG)_14_
36B4‐Forward	CAGCAAGTGGGAAGGTGTAATCC
36B4‐Reverse	CCCATTCTATCATCAACGGGTACAA
36B4 standard	CAGCAAGTGGGAAGGTGTAATCCGTCTCCACAGACAAGGCCAG GACTCGTTTGTACCCGTTGATGATAGAATGGG

### 4.3 Senescence‐Associated β‐Galactosidase (SA‐β‐Gal) Activity Assay

Cells were seeded in six‐well plates and grown until they reached 80% confluence. Cells were washed with PBS pH 6 and fixed with 3% (w/v) paraformaldehyde/2% (w/v) sucrose in PBS for 10 min at RT. After rinsing with PBS pH 6, coverslips were stained with freshly prepared SA‐β‐gal solution for 16–24 h at 37°C, and sealed in parafilm and aluminum foil. After incubation, cells were washed twice with PBS for 2 min, briefly rinsed with DMSO to remove salt crystals, and washed with PBS for 5 min. Coverslips were mounted on slides with Fluoromount‐G mounting medium (Electron Microscopy Sciences, Cat. No. 17984). Images were taken with an iPhone 8 on a Zeiss phase contrast microscope.

### 4.4 Telomere Length Analysis by TRF Assay

Telomeres were examined using in‐gel hybridization and were visualized using a radiolabeled (TTAGGG)_x_ telomeric DNA probe labeled with Klenow polymerase and [α‐^32^P] dGTP (3000 Ci mmol^−1^; Perkin‐Elmer) as described in (Tsang et al. 2012).

### 4.5 Transcriptome Analyses

Young and old BJ fibroblast cells and strains expressing the three hTERT variants were grown in triplicate, and log phase cells were harvested. RNA was isolated using Trizol (Invitrogen), purified using the RNeasy Mini Kit (Qiagen), and purity and concentration of RNA were assessed initially using a Nanodrop ND‐1000 (Thermo Scientific). Total RNA sample integrity was assessed via TapeStation and fluorescent quantification assays. Samples with RNA integrity number (RIN) scores greater than 9 were used. Library preparation was performed with 800 ng inputs using the New England Biolabs NEBNext Ultra II directional RNA kit with poly A capture module. Finished libraries were assessed via Kapa q‐PCR assay. Sequencing was performed on an Illumina NovaSeq6000 instrument with 2 × 50 bp read lengths targeting 43 M read pairs before analysis. Quality metrics were performed using the Sequence Analysis Viewer (SAV) software provided by Illumina. Subsequently, base calling and demultiplexing were performed using the CASAVA 1.8.1 software pipeline with default settings. These steps result in one or several fastq files per sample that contain short reads and base qualities in Sanger format. Short read quality was examined with FastQC (http://www.bioinformatics.bbsrc.ac.uk/projects/fastqc/) software. Adaptors were trimmed using cutadapt software (http://code.google.com/p/cutadapt/), and another FastQC quality check was performed. Ultimately, short reads were aligned to the human genome (hg38) using the Bowtie aligner, and the counts of reads that aligned to features of interest were then fed into the program Cufflinks to determine differential gene expression and perform statistical analysis. Venn diagrams were generated using the Venn Diagram package from the Comprehensive R Archive Network (CRAN).

### 4.6 STELA Assays

DNA was isolated from two independent cultures, each of young, old, WT hTERT‐expressing, V144M mutant hTERT‐expressing, and R865C mutant hTERT‐expressing BJ fibroblasts. Samples were coded and examined by STELA in a blind experimental protocol. DNA was quantified in triplicate by Hoechst 33258 fluorometry (Promega). STELA was carried out at the 17p telomere as described previously (Baird et al. 2003; Capper et al. 2007). For each sample, 1 μM of the Telorette2 linker was added to 10 ng of purified gDNA in a final volume of 40 μL of 10 mM Tris–HCl. Six PCR reactions were performed for each test DNA in volumes of 10 μL, incorporating 1 μL of the DNA/Telorette‐2 mix and 0.5 μM of the 17pseq1rev and Teltail primers in 75 mM Tris–HCl pH 8.8, 20 mM (NH_4_)_2_SO_4_, 0.01% Tween‐20, and 1.5 mM MgCl_2_, with 0.5 U of a 10:1 mixture of Taq (ABGene) and Pwo polymerase (Roche Molecular Biochemicals).

Oligonucleotides used were as follows:

17pseq1rev: GAATCCACGGATTGCTTTGTGTAC.

Teltail: TGCTCCGTGCATCTGGCATC.

Telorette2: TGCTCCGTGCATCTGGCATCTAACCCT.

The reactions were subjected to 21 cycles of 94°C for 20 s, 59°C for 30 s, and 68°C for 8 min using a Tetrad2 Thermal Cycler (Bio‐Rad). DNA fragments were resolved using 0.5% Tris‐acetate‐EDTA agarose gel electrophoresis and identified via Southern hybridization using a random‐primed α‐^33^P‐labeled (Perkin‐Elmer) TTAGGG repeat probe, together with probes specific for the 1 kb (Stratagene) and 2.5 kb molecular weight markers (Bio‐Rad). Hybridized fragments were detected using a Typhoon FLA 9500 Phosphorimager (GE Healthcare). The molecular weights of the DNA fragments were calculated using a Phoretix 1D Quantifier (Nonlinear Dynamics).

### 4.7 Metaphase Spreads, Fluorescence In Situ Hybridization (FISH) Staining, Immunoblotting

Metaphase spreads, FISH, and immunoblotting were performed as described (Adam et al. 2019).

### 4.8 Immunofluorescence (IF) and IF‐FISH Assays

Cells were grown to 80% confluence on coverslips in 6‐, 12‐, or 24‐well plates. Next, cells were washed with PBS and fixed with 3% (w/v) paraformaldehyde/2% (w/v) sucrose in PBS for 10 min at RT. Cells were washed three times with PBS for 5 min at RT and permeabilized for 4 min with 0.5% (v/v) Triton X‐100 in PBS at RT. After a brief wash with PBS, cells were preblocked at RT for 10 min with 2% (w/v) BSA in PBS. Next, cells were blocked at RT for 30 min with 10% (v/v) normal goat serum in PBS. After rinsing with PBS, the cells were incubated with primary antibody for 53BP1 (1:1000, Abcam, ab21083), γH2AX (1:1000, Abcam, ab26350), or Lamin A/C (1:1000, Santa Cruz, sc7292) for 60 min at RT. The cells were washed three times with PBS, followed by three times 5 min with PBS, and incubated with secondary antibody for Alexa‐488 (1:600, Invitrogen, A11029), Alexa‐568 (1:600, Invitrogen, A11011), or Alexa‐647 (1:600, Invitrogen, A21246) for 25–30 min at RT. Primary and secondary antibodies were diluted in 2% (w/v) BSA in PBS and centrifuged before use (5 min, 13,000 rpm, 4°C). For IF‐FISH, coverslips were fixed with 3% (w/v) paraformaldehyde/2% (w/v) sucrose in PBS for 30 min at RT, followed by the direct FISH protocol. Cells were counterstained with PBS containing DAPI (Millipore Sigma, 1:10.000) and mounted on slides with ProLong Diamond Antifade Mounting solution (Thermo Fisher Scientific, Cat. No. P36970).

### 4.9 Antibodies Used in Western Blotting

For western blotting, all antibodies were used at a dilution of 1:1000 unless otherwise noted and were from the following suppliers:

53BP1 (Abcam, ab21083, rabbit).

α‐Tubulin (Novus Biologicals, NB100‐690, mouse).

ATM (Abcam, ab32420, rabbit).

ATM (p‐S1981) (Abcam, ab81292, rabbit).

β‐actin (Thermo scientific, AM4302, mouse).

CDC20 (Proteintech, 10252‐1‐AP, polyclonal Rabbit).

FBXO5 (Emi1; Abcam, EPR15320 (ab187144), Monoclonal Rabbit).

γH2AX (Abcam, ab26350, mouse).

Lamin A/C (Santa Cruz, sc7292, mouse).

p16 (BD Pharmingen, No. 554079, mouse).

p53 (Calbiochem, OP43, mouse).

p53 (p‐S15) (Abcam, ab1431, rabbit).

Rb (Santa Cruz, sc73598, mouse).

SIAH1(Abcam, ab2237, Goat).

Telomerase catalytic subunit antibody (Rockland, 600‐401‐252S, Rabbit).

TNFR1(Abcam, ab19139, Rabbit, 1:1000).

TNFR2 (Cell Signaling, CST No. 72337, Rabbit, 1:1000).

TRF1 (Abcam, ab129177, Rabbit).

TRF2 (Novus Biologicals, NB110‐57130, Rabbit).

### 4.10 Confocal and SIM Image Acquisition

For confocal acquisition, a 63×/1.47 NA oil objective was used on a Quorum Diskovery Flex multimodal microscope using the spinning disk confocal modality. For 3D SIM acquisition, a 100×/1.42 NA oil objective on a DeltaVision OMX SR imaging system was used.

### 4.11 STORM Imaging, Reconstruction, and SMLM Analysis

For direct STORM (dSTORM), telomeres were stained with 100 nM TelC‐Alexa647 conjugated PNA‐probe (PNA Bio, F1013), and the coverslips were stored in 1× PBS at 4°C wrapped in aluminum foil until ready to image.

### 4.12 Statistical Analysis

All statistical analyses were performed in GraphPad Prism 8.2.1. Measurements were taken from distinct samples, and three independent biological replicates (*n* = 3) were measured for each experiment, unless specified otherwise. To determine statistical significance, a student's *t*‐test was used to compare measurements between young and senescent cells. A one‐way ANOVA was performed to compare measurements between young, senescent, WT‐, R865C‐, and V144M‐hTERT cells. The specific statistical tests and significant *p* values are indicated in the figure legends.

## Author Contributions

N.A. performed the majority of the experiments, analyzed the images, and wrote the first draft of the manuscript. Y.Y. grew cells and performed the RNAseq experiments, western blotting, and indirect immunofluorescence experiments on IPF and DKC cells. M.D. analyzed RNAseq data for SASP, TP53 gene targets, DDR, and HR genes; N.S.Y.T. grew cells and harvested DNA for STELA and helped with the dSTORM reconstruction; H.K. assisted with western blotting; J.G. and N.T. assisted with the dSTORM imaging and coding in RStudio and Python; P.M.K.G. completed initial bioinformatics analyses of RNAseq data; K.V.V.W. performed statistical analyses; D.M.B. obtained financial support and coordinated STELA analyses; S.S. performed STELA assays; C.K.G. and S.A.S. provided background information and cell samples for IPF and DC patients, respectively; and A.A.G., T.L.B., and K.R. directed the study, obtained financial support, assisted in study design, and manuscript writing.

## Conflicts of Interest

The authors declare no conflicts of interest.

## Supporting information


Figure S1.

Figure S2.

Figure S3.

Figure S4.

Figure S5.

Figure S6.

Figure S7.

Figure S8.

Figure S9.

Table S1.

## Data Availability

Data availability statementTranscriptomic and additional data that support the findings of this study are available from the corresponding authors upon reasonable request.
