# Supplementary figures and images for: hTERT Increases TRF2 to Induce Telomere Compaction and Extend Cell Replicative Lifespan
Source: Aging Cell. 2025 May 15;24(8):e70105. doi: 10.1111/acel.70105 (PMC12341801; doi:10.1111/acel.70105)

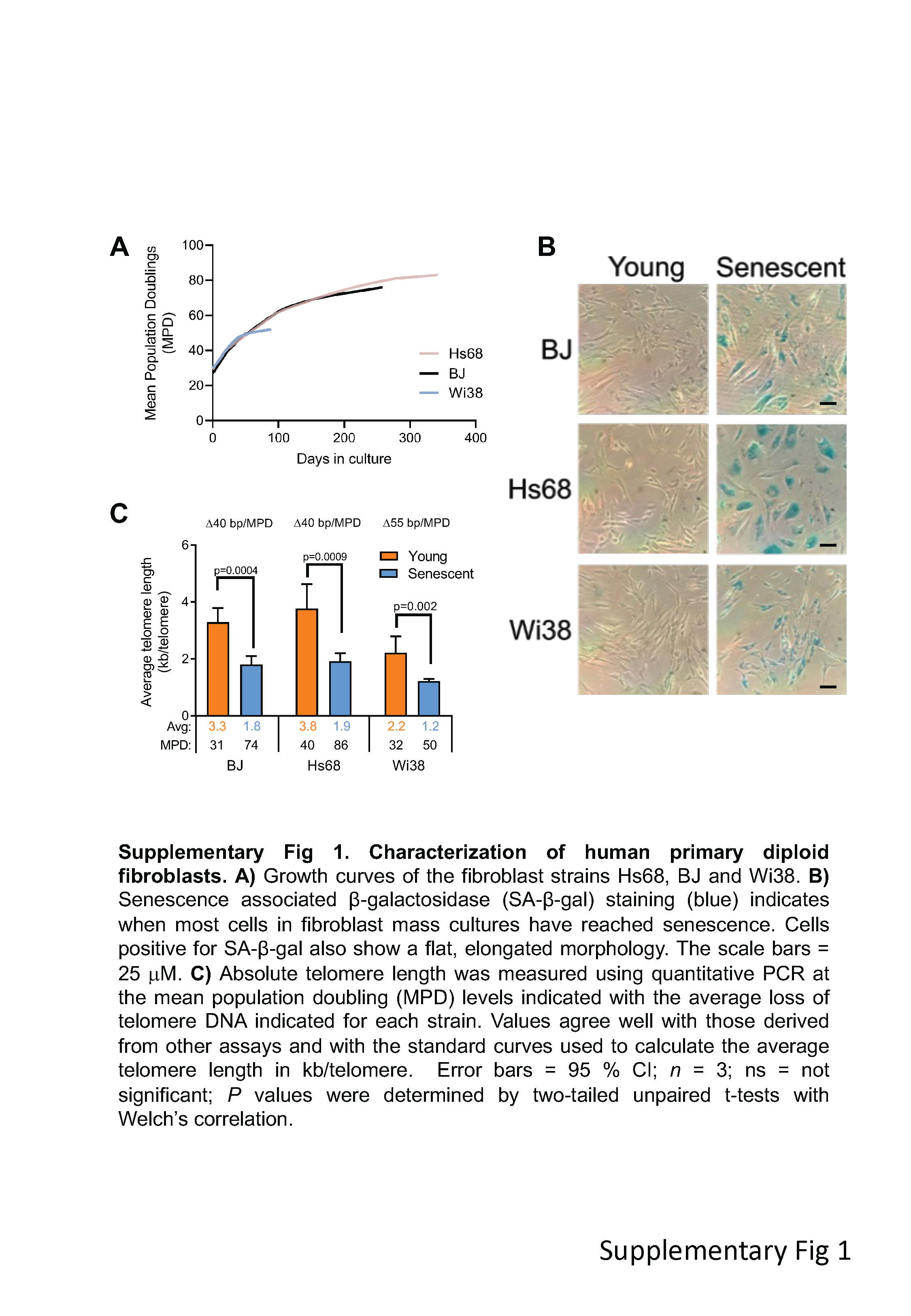

Supplement: Supplementary file 1 — Figure S1. [file ACEL-24-e70105-s002.tiff]

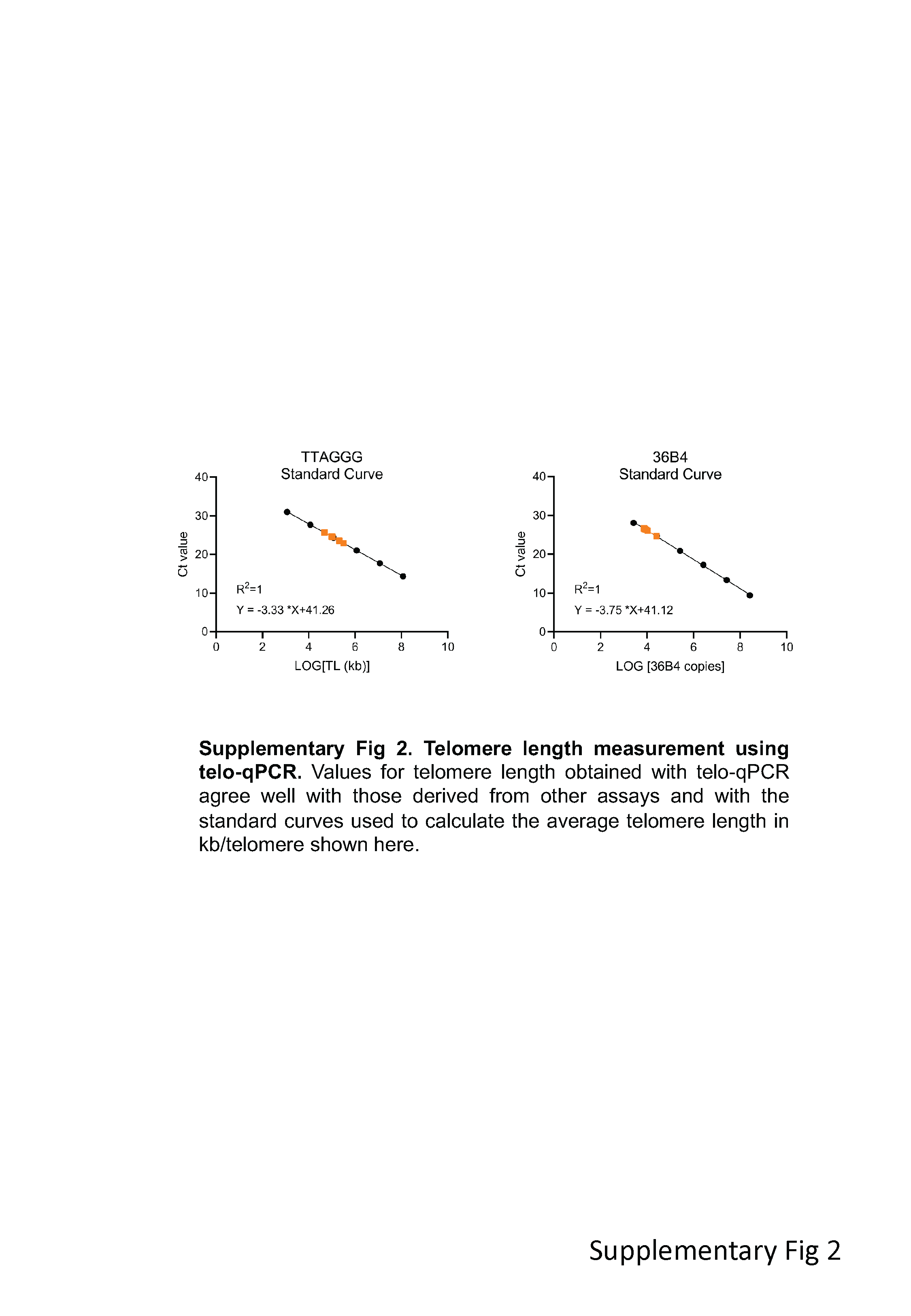

Supplement: Supplementary file 2 — Figure S2. [file ACEL-24-e70105-s006.tiff]

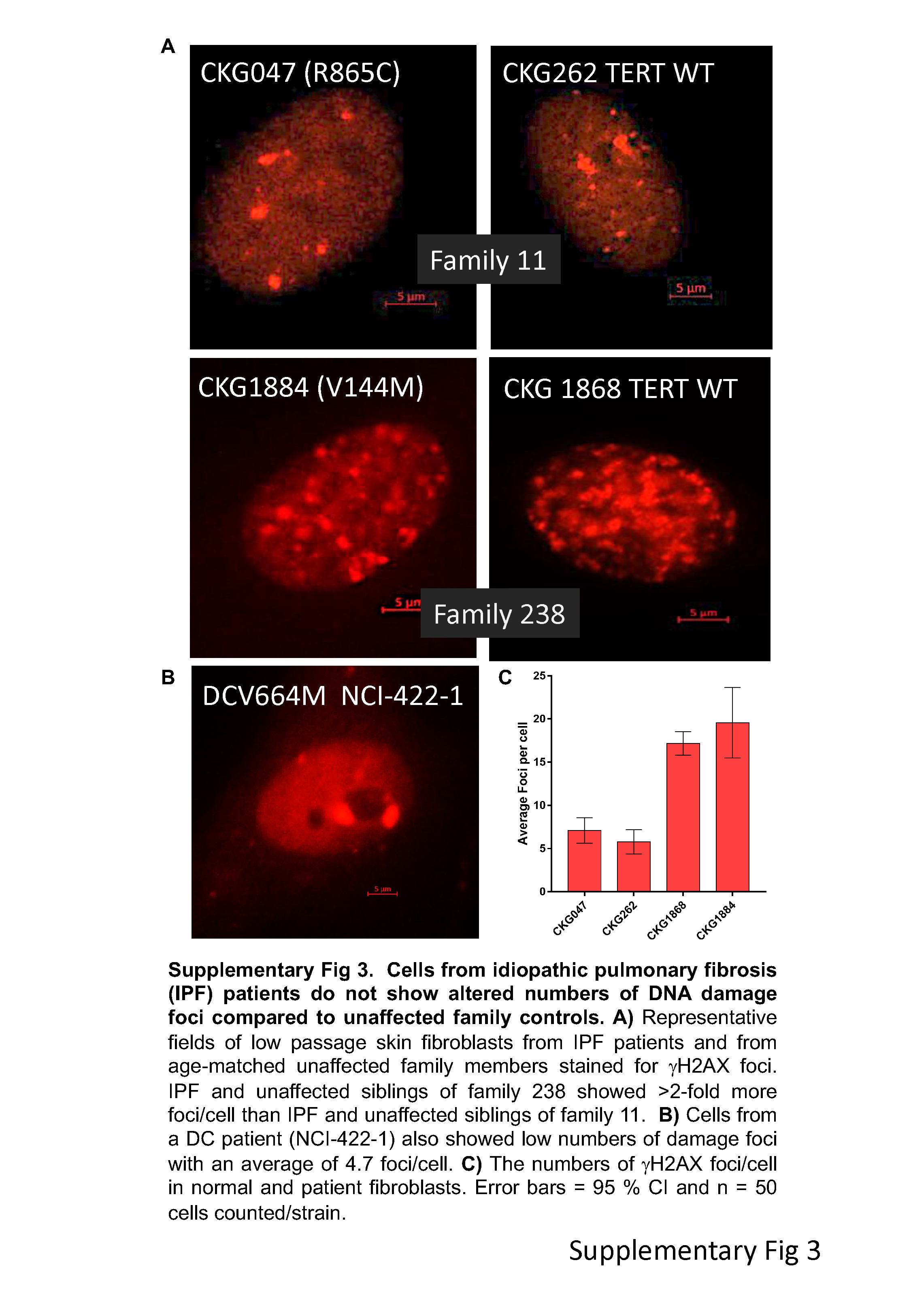

Supplement: Supplementary file 3 — Figure S3. [file ACEL-24-e70105-s004.tiff]

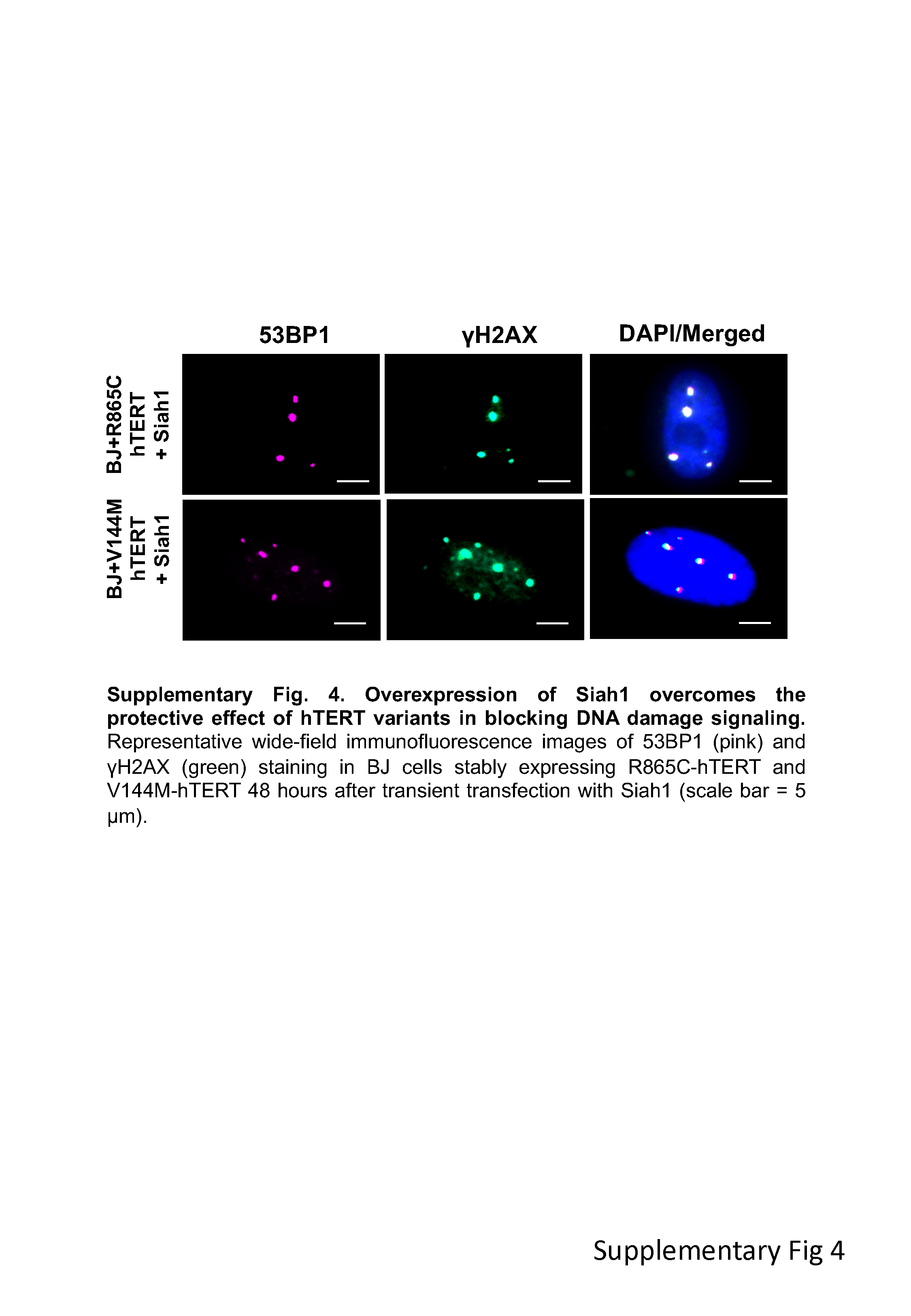

Supplement: Supplementary file 4 — Figure S4. [file ACEL-24-e70105-s005.tiff]

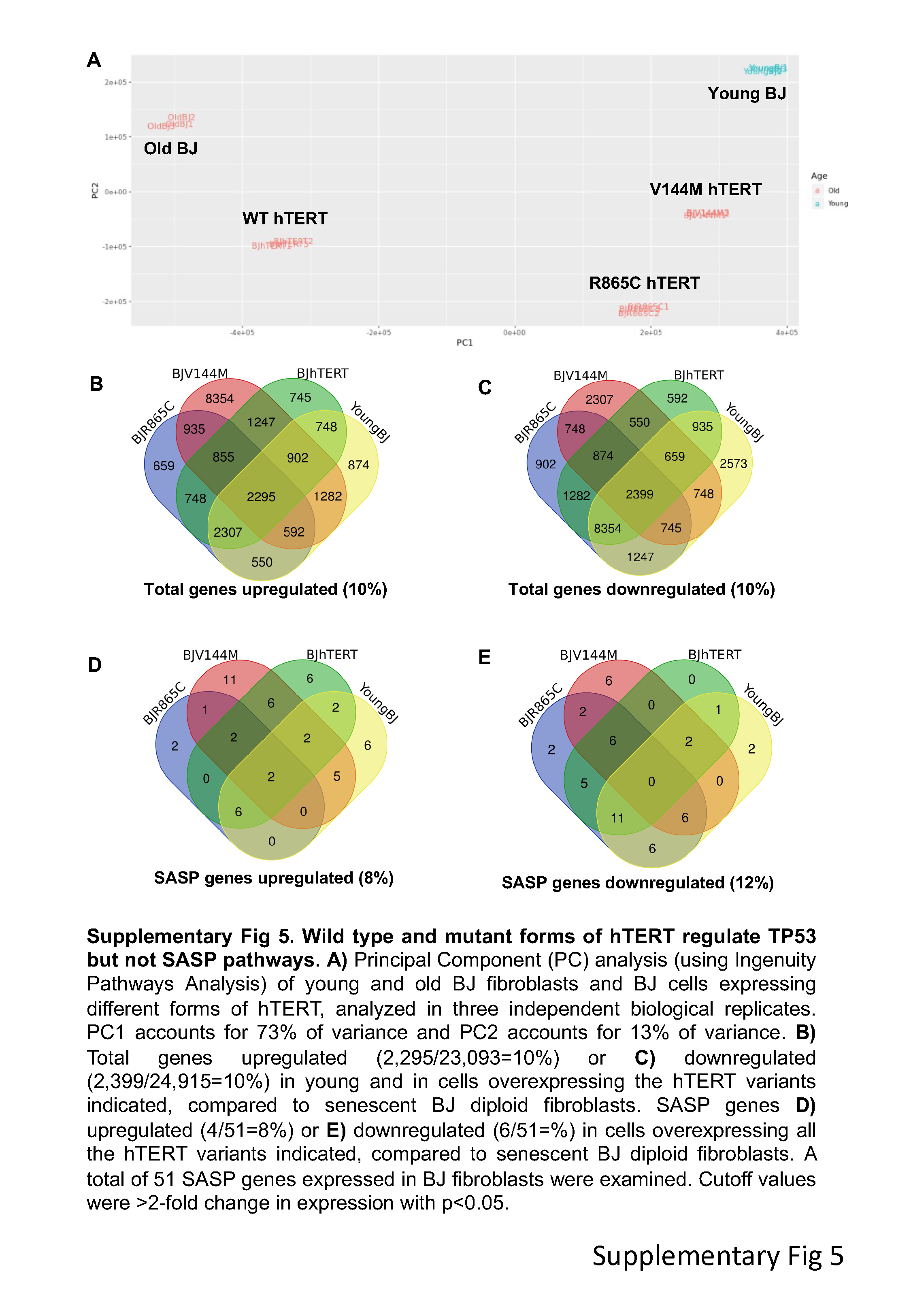

Supplement: Supplementary file 5 — Figure S5. [file ACEL-24-e70105-s001.zip › acel70105-sup-0005-FigureS5.tiff]

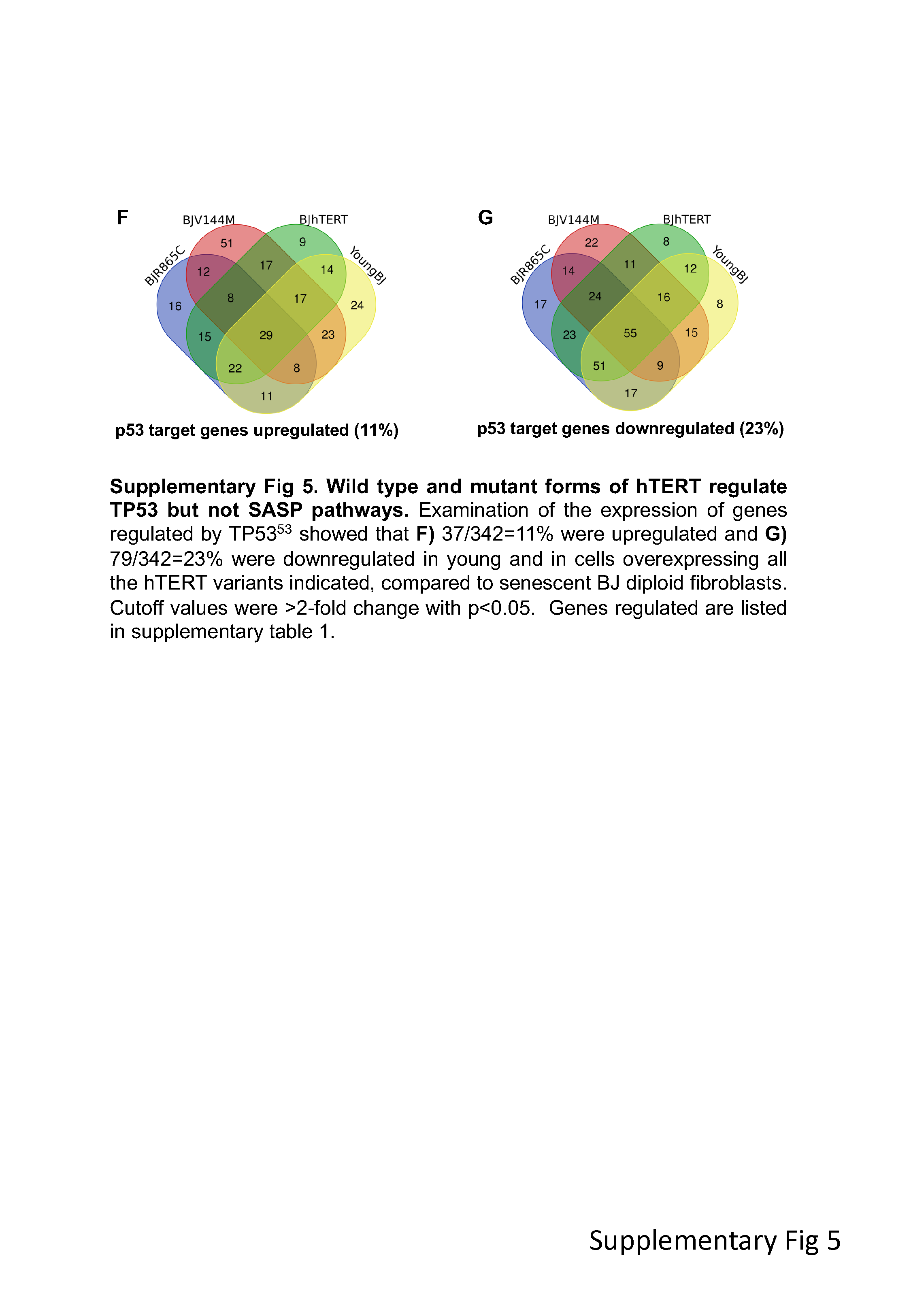

Supplement: Supplementary file 5 — Figure S5. [file ACEL-24-e70105-s001.zip › acel70105-sup-0006-FigureS5.tiff]

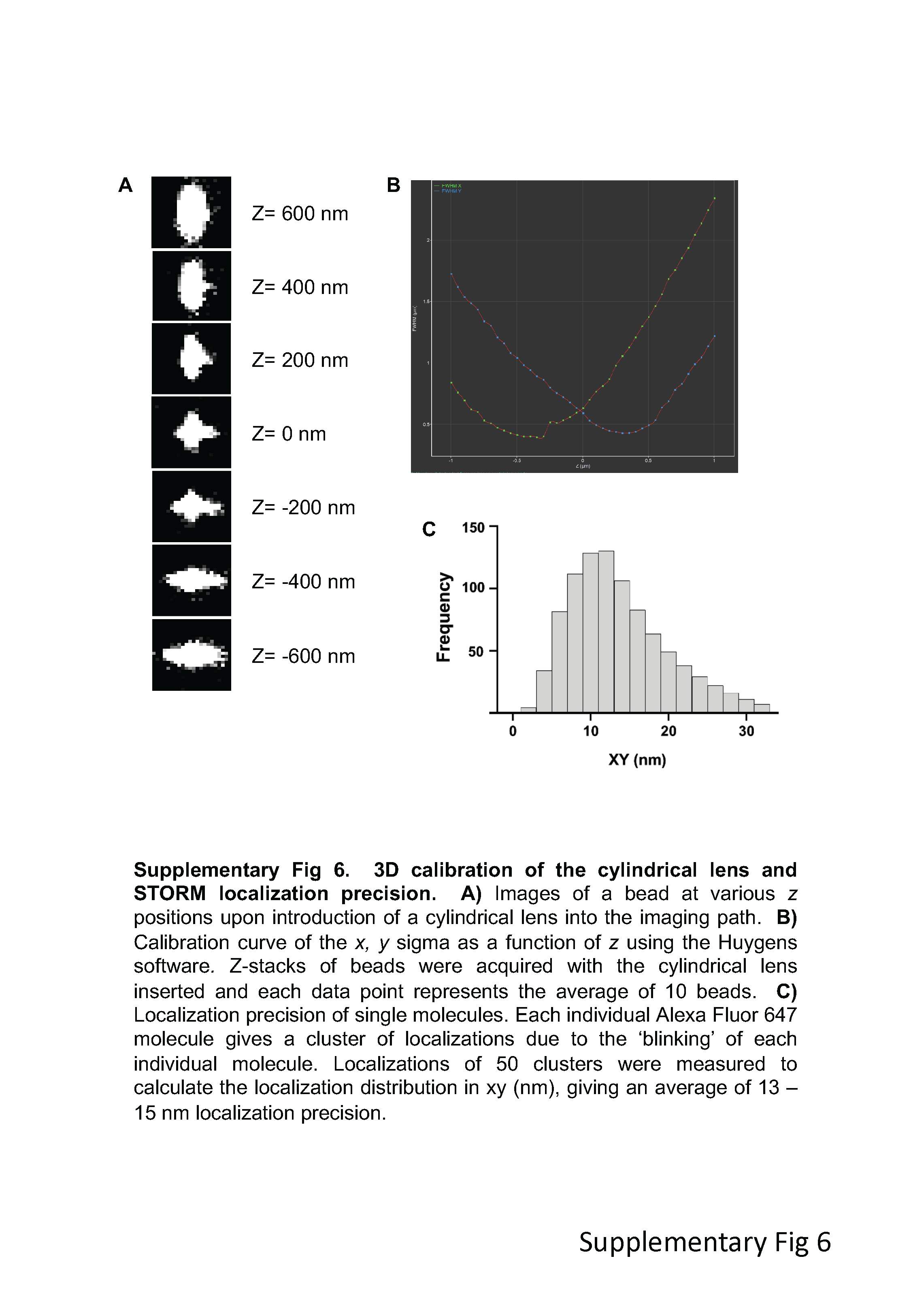

Supplement: Supplementary file 6 — Figure S6. [file ACEL-24-e70105-s009.tiff]

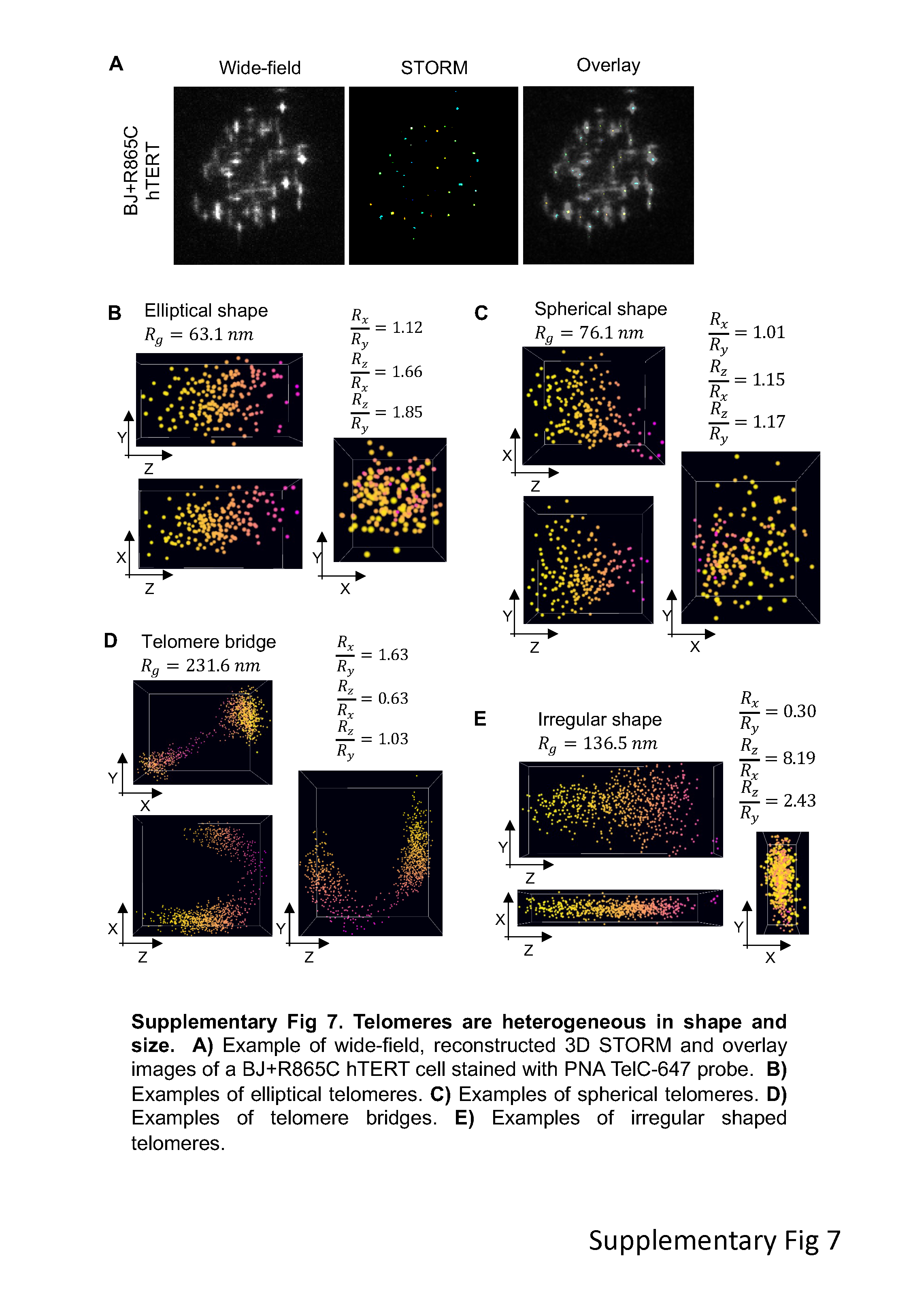

Supplement: Supplementary file 7 — Figure S7. [file ACEL-24-e70105-s010.tiff]

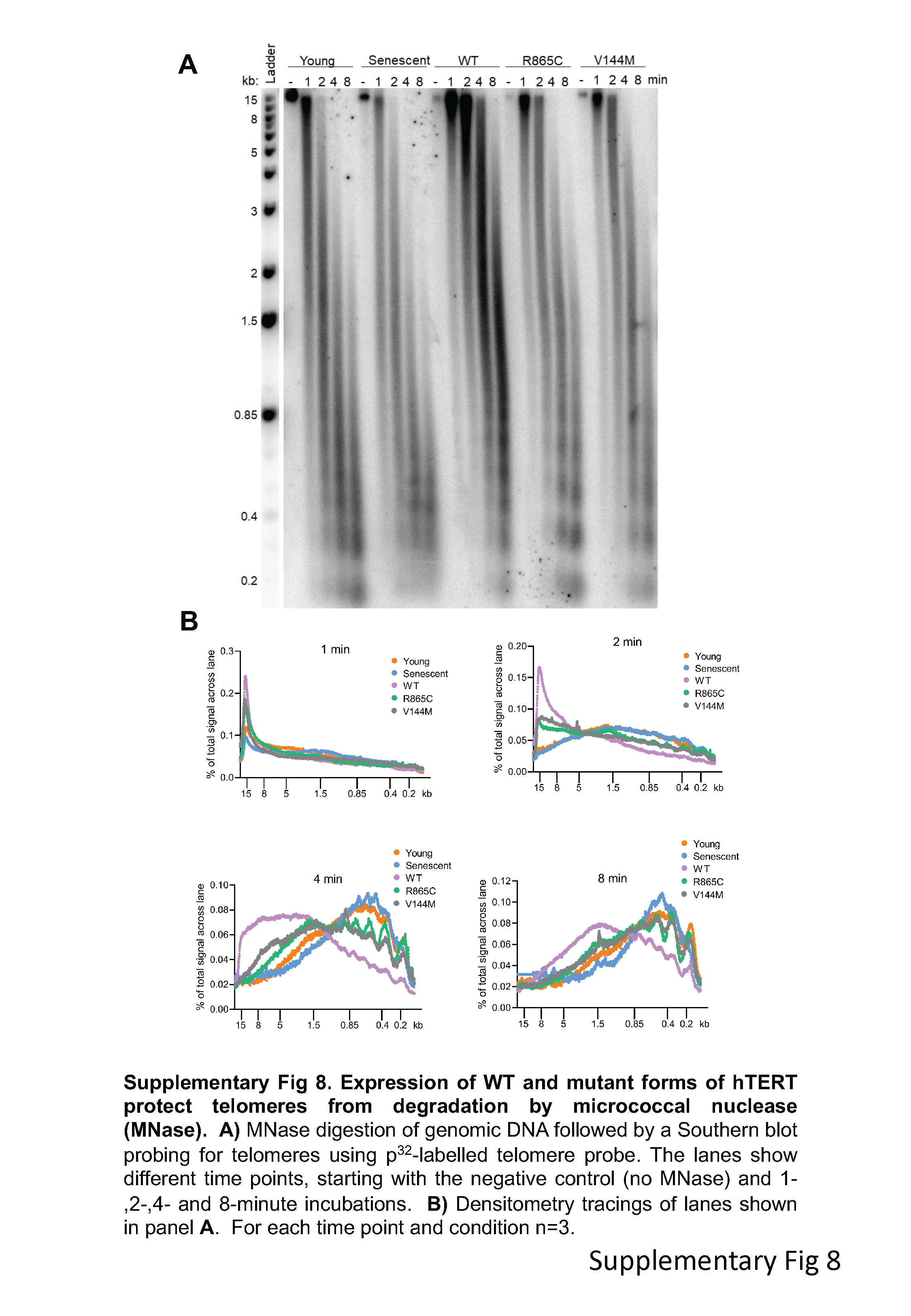

Supplement: Supplementary file 8 — Figure S8. [file ACEL-24-e70105-s008.tiff]

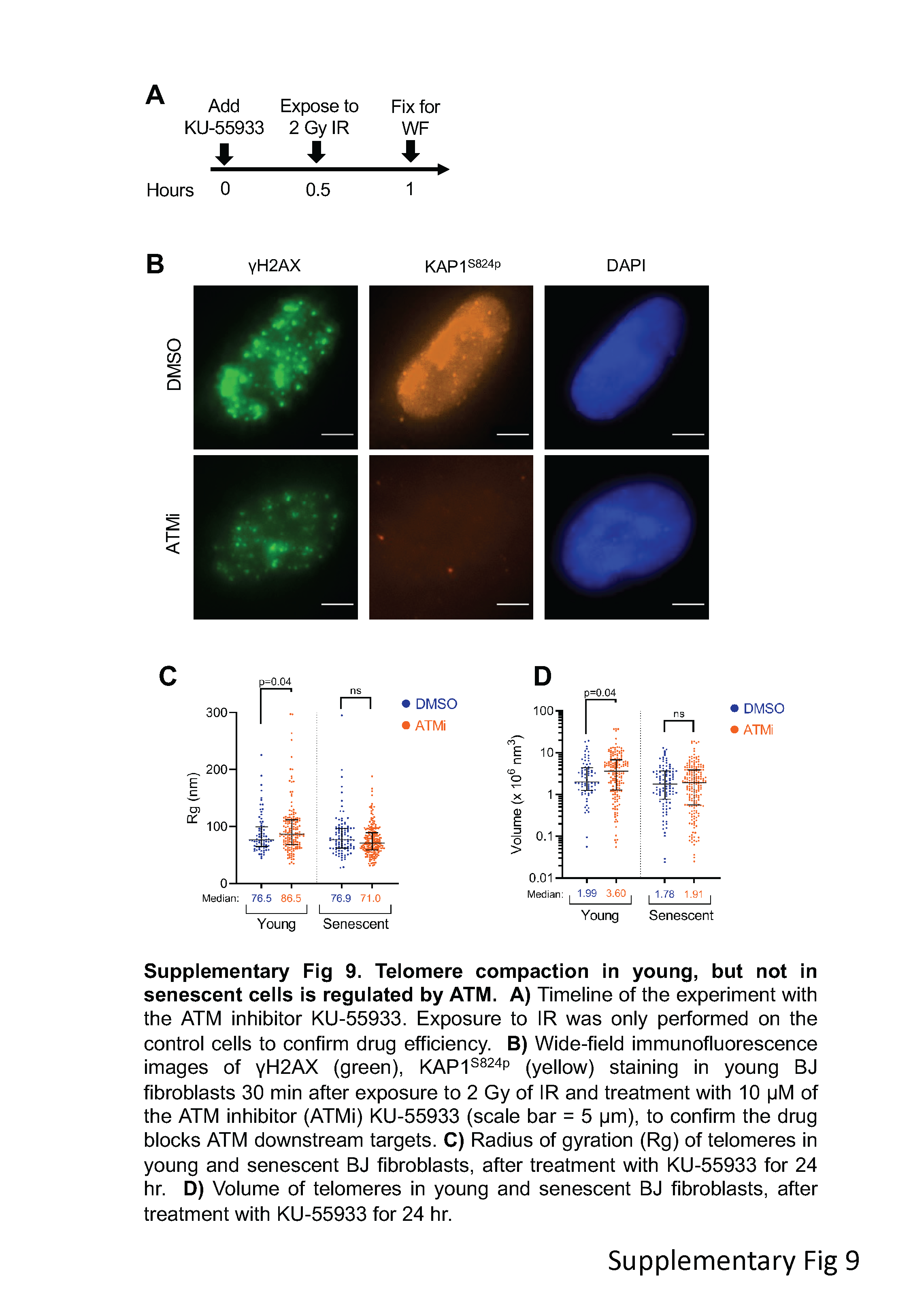

Supplement: Supplementary file 9 — Figure S9. [file ACEL-24-e70105-s003.tiff]

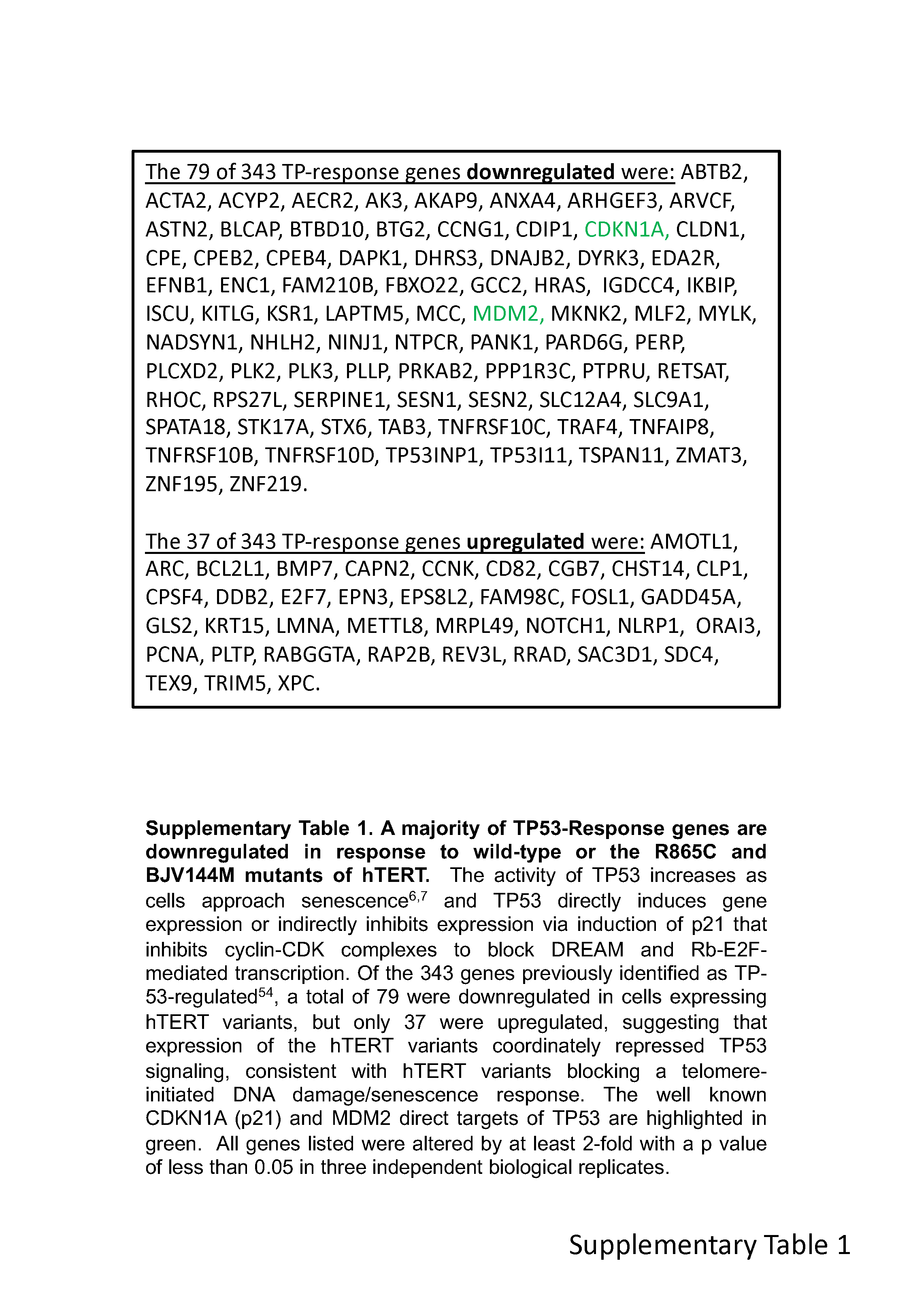

Supplement: Supplementary file 10 — Table S1. [file ACEL-24-e70105-s007.tiff]
